# Transcription factors enhancing synthesis of recombinant proteins and resistance to stress in *Yarrowia lipolytica*

**DOI:** 10.1007/s00253-023-12607-z

**Published:** 2023-06-15

**Authors:** Maria Gorczyca, Jean-Marc Nicaud, Ewelina Celińska

**Affiliations:** 1 Department of Biotechnology and Food Microbiology, Poznan University of Life Sciences, 60-637 Poznań, Poland; 2 Université Paris-Saclay, INRAE, AgroParisTech, Micalis Institute, 78350 Jouy-en-Josas, France

**Keywords:** Stress response, Environmental stress factor, Heterologous protein, Yeast, Transcription factor, Protein expression platform

## Abstract

**Abstract:**

Resistance to environmental stress and synthesis of recombinant proteins (r-Prots) are both complex, strongly interconnected biological traits relying on orchestrated contribution of multiple genes. This, in turn, makes their engineering a challenging task. One of the possible strategies is to modify the operation of transcription factors (TFs) associated with these complex traits. The aim of this study was to examine the potential implications of selected five TFs (*HSF1-YALI0E13948g*, *GZF1-YALI0D20482g*, *CRF1-YALI0B08206g*, *SKN7-YALI0D14520g*, and *YAP-like-YALI0D07744g*) in stress resistance and/or r-Prot synthesis in *Yarrowia lipolytica*. The selected TFs were over-expressed or deleted (OE/KO) in a host strain synthesizing a reporter r-Prot. The strains were subjected to phenotype screening under different environmental conditions (pH, oxygen availability, temperature, and osmolality), and the obtained data processing was assisted by mathematical modeling. The results demonstrated that growth and the r-Prot yields under specific conditions can be significantly increased or decreased due to the TFs’ engineering. Environmental factors “awakening” individual TFs were indicated, and their contribution was mathematically described. For example, OE of Yap-like TF was proven to alleviate growth retardation under high pH, while Gzf1 and Hsf1 were shown to serve as universal enhancers of r-Prot production in *Y. lipolytica*. On the other hand, KO of *SKN7* and *HSF1* disabled growth under hyperosmotic stress. This research demonstrates the usefulness of the TFs engineering approach in the manipulation of complex traits and evidences newly identified functions of the studied TFs.

**Key points:**

*• Function and implication in complex traits of 5 TFs in Y. lipolytica were studied.*

*• Gzf1 and Hsf1 are the universal r-Prots synthesis enhancers in Y. lipolytica.*

*• Yap-like TF’s activity is pH-dependent; Skn7 and Hsf1 act in osmostress response.*

**Supplementary Information:**

The online version contains supplementary material available at 10.1007/s00253-023-12607-z.

## Introduction

Recombinant protein (r-Prot) synthesis and stress response are both complex and strongly interconnected biological processes of great industrial importance. Environmental stress factors strongly impact the overall performance of a microbial producer cell, including its growth rate, metabolic activity, and capacity to produce r-Prots. On the other hand, high-level synthesis of r-Prots modulates the cell’s ability to cope with unfavorable environmental conditions. Due to the traits’ complexity, usually, multiple genes must be fine-tuned to achieve their effective engineering.

The molecular background of the environmental stress response has been extensively studied in the model yeast species – *Saccharomyces cerevisiae* (e.g., Gasch et al. 2000; Gasch and Werner-Washburne 2002; Gasch 2007; Hou et al. 2013; Liu et al. 2013), unrevealing the major molecular players of this process (Craig et al. 1993; Verghese et al. 2012). In addition, it has been evidenced that excessive synthesis of r-Prots leads to endogenous stress, awakening massive cellular response (Mattanovich et al. 2004; Matsumoto et al. 2005; Tyo et al. 2012; Hou et al. 2014; de Ruijter and Frey 2015). Further detailed molecular studies revealed substantial overlap between such environmental and endogenous stress responses (Hahn et al. 2004; Guyot et al. 2005; Verghese et al. 2012; Hou et al. 2013). For example, it was shown that 3% of the *S. cerevisiae* genome was induced by heat shock, out of which 25% was constituted by the proteins involved in translation and polypeptides’ secretion (Hahn and Thiele 2004; Hahn et al. 2004). That observation was later supported by evidence that synthetic induction of the heat shock response improved r-Prot synthesis in *S. cerevisiae* (Hou et al. 2013).

*Yarrowia lipolytica* is a dimorphic yeast species that has gained significant interest as an r-Prots production platform (Nicaud et al. 2002; Groenewald et al. 2014; Madzak 2015, 2018, 2021). Thanks to multiple comprehensive investigations (e.g., Boisramé et al. 1999, 2002; Kabani et al. 2000; Swennen and Beckerich 2007; Babour et al. 2008; Swennen et al. 2010; Madzak and Beckerich 2013; Korpys-Woźniak et al. 2020; Korpys-Woźniak and Celińska 2021), reviewed in Celińska and Nicaud (2019), many mechanistic and functional details of r-Prots synthesis in this species have been revealed. As an industrial workhorse, *Y. lipolytica* is known to efficiently fight against different environmental threats. The molecular background of this resistance/stress response was analyzed in multiple studies (e.g., Yang et al. 2015; Pomraning et al. 2016; Walker et al. 2019; Cogo et al. 2020; Kolhe et al. 2021; Kubiak-Szymendera et al. 2021; Lesage et al. 2021; Sekova et al. 2021), reviewed in Celińska (2022).

Research addressing the mutual impact of r-Prot synthesis and resistance to environmental stress factors in *Y. lipolytica* is still very scarce. Sassi et al. (2017) studied the impact of pH and cell morphology on r-Prot synthesis in *Y. lipolytica* and demonstrated that only the former significantly contributes to the r-Prot yields. Kubiak et al. (2021) optimized conditions of thermal treatment that promote the synthesis of r-Prots in bioreactor cultures, showing that decreased temperature favors the synthesis of r-Prots. Further studies demonstrated that the treatment with decreased temperature specifically relieves secretion of the r-Prots in *Y. lipolytica* (Korpys-Woźniak et al. 2021). The molecular background of that phenomenon was investigated by global proteomics and gene expression analysis (Kubiak-Szymendera et al. 2021). The same report provided data on the impact of hyperosmolality (Osm) on the r-Prot synthesis and ultimately settled that it is not favorable for secretory r-Prots production by *Y. lipolytica* (Kubiak-Szymendera et al. 2021). The mechanisms underlying the globally decreased synthesis of proteins under Osm in *Y. lipolytica* were pointed out in that report. The impact of the key factor limiting *Y. lipolytica* performance in the industry, namely oxygen availability (pO_2_), on r-Prots synthesis, was also studied (Gorczyca et al. 2020, 2022). The former study showed that limited pO_2_ negatively impacts all – the rate of transcription, translation, and secretion of secretory r-Prots (Gorczyca et al. 2020). It was also established that the r-Prot synthesis rate is not directly related to the rate of biomass growth in *Y. lipolytica*, and the former can be limited when the latter is stably maintained. In the following study, it was demonstrated and quantitatively expressed that the metabolic burden caused by the over-synthesis of two complex r-Prots in *Y. lipolytica* significantly increased substrate consumption, even at a reduced growth rate (Gorczyca et al. 2022). In addition, it was evidenced that the high metabolic burden caused by r-Prots synthesis has a significant impact on the host’s stress resistance (pO_2_ and pH).

The complex traits can be efficiently engineered by pursuing an adaptive laboratory evolution approach followed by reverse engineering (Portnoy et al. 2011; Dragosits and Mattanovich 2013; Winkler and Kao 2014; Mans et al. 2018) or by applying a directed genetic engineering strategy, but targeting molecular identities operating at a higher level of the molecular events, like signaling cascades or transcription factors (TFs). For example, overexpression of the *HAC1* gene (TF implicated in the regulation of ER (endoplasmic reticulum)-resident events and restoring ER homeostasis) improved r-Prots synthesis in *S. cerevisiae* (Duan et al. 2019), *Pichia pastoris* (Guerfal et al. 2010), and *Y. lipolytica* (Korpys-Woźniak et al. 2021). Titers of different r-Prots were increased by nearly threefold upon overexpression (OE) of different translation initiation factors in *P. pastoris* (Staudacher et al. 2022), and by over fivefold when the polypeptides translocation step was engineered by “pushing” the flux on the cytosolic side and “pulling” on the ER side (Zahrl et al. 2022). Secretory r-Prots production was significantly enhanced in *S. cerevisiae* upon co-overexpression (co-OE) of *HSF1* (heat shock factor) which encodes the key regulator of heat shock response (Hou et al. 2013). Continuous activation of heat shock response by co-OE of a mutant *HSF1*-R206S triggered increased production of native proteins and r-Prots. Co-OE of *HAP1* (responsible for aerobic metabolism and activation of oxidative stress-responsive) mitigated the negative effects of oxidative stress caused by intensive protein folding and hence increased the r-Prots production capacity in *S. cerevisiae* (Martínez et al. 2016). Very recently, synthetic activation of the general stress response TF Msn4 (and its synthetic version synMsn4, alone or in combination) triggered over fourfold enhancement in r-Prot production (Zahrl et al. 2023). Nevertheless, only a limited number of TFs have been studied in yeast in the context of r-Prots synthesis and the associated stress resistance yet.

While *Y. lipolytica* is a well-recognized r-Prots production platform, TFs implicated in stress response or protein synthesis are still not well described. Most of the literature reports refer to the role of an individual TF in the regulation of another complex trait – dimorphic transition. Involvement in the morphological changes has been proven for TF Msn2 (Pomraning et al. 2018), Yap-like encoded by *YALI0D07744g* (Morales-Vargas et al. 2012), Znc1 (Martinez-Vazquez et al. 2013), Bmh1 (Hurtado and Rachubinski 2002), Mhy1 (Hurtado and Rachubinski 1999; Wu et al. 2019), as well as for Hoy1 (Torres-Guzmán and Domínguez 1997). In addition, TF Msn4 was shown to regulate tolerance to acid-induced stress (Wu et al. 2019). Several reports addressed the issue of TF-driven regulation of lipid accumulation in *Y. lipolytica* (Beopoulos et al. 2009; Nicaud 2012). In this regard, functional studies on individual knockout (KO) genotypes, *Δpor1* (Poopanitpan et al. 2010), *Δmig1* (Wang et al. 2013), *Δyas1* and *Δyas2* (Endoh-Yamagami et al. 2007), *Δyas3* (Hirakawa et al. 2009), and *Δgzf2* and *Δgzf3* (Pomraning et al. 2018), were reported. The impact of constitutive overexpression (OE) of one hundred twenty-five TFs on lipids accumulation in *Y. lipolytica* has been studied through a high-throughput screening approach (Leplat et al. 2018).

The aim of this study was to examine the potential implications of selected five TFs in stress resistance and/or r-Prot synthesis in *Y. lipolytica*. The adopted strategy relied on the co-OE or KO of selected TFs in a host strain synthesizing r-Prot. The strains were subjected to extensive phenotype screening under different environmental conditions, and the data processing was assisted by mathematical modeling. It was presumed that apart from genetic engineering of the TFs-encoding genes, implementation of the environmental perturbations will further manipulate the activation status of the TFs. A practical goal was to evaluate this approach as a rationale engineering strategy to enhance stress resistance and/or r-Prot synthesis in *Y. lipolytica*.

## Materials and methods

### Microbial strains and basic media

All the strains used in this study are listed in Supplementary Tables S1A (*Y. lipolytica* – final recipient of genetic constructions) and S1B (*Escherichia coli* – cloning and plasmid propagation). The yeast strains are derivatives of the JMY2566 strain, engineered in the *URA3* locus to contain a zeta-docking platform and OE, an intracellular fluorescent (FL) reporter protein (RedStarII) under the p*TEF* promoter (Leplat et al. 2015). JMY2810 strain is a prototrophic derivative of JMY2566 transformed with an empty *URA3* cassette. Strains “OE-TFx” bear an expression cassette encoding one of the selected TFs under the control of the p*TEF* constitutive promoter, integrated at the zeta platform (Leplat et al. 2018). Strains “KO-TFx” were constructed on the platform of the JMY2810 strain by disrupting the indicated locus encoding selected TF with a cassette bearing a *NATr* (nourseothricin) gene flanked with regulatory elements, and approximately 1 kbp of a complementary region on each side of the cassette.

The yeast strains were routinely maintained at 28 °C in rich YPD (g L^−1^: yeast extract, 5 (BTL, Łódź, Poland); peptone, 10 (BTL); glucose, 20 (POCH, Gliwice, Poland); solidified with agar, 15 (BTL)) or in minimal YNB medium (g L^−1^: glucose, 10 (POCH); yeast nitrogen base, 1.7 (Sigma-Aldrich, St. Louis, USA); ammonium sulfate, 5 (POCH); solidified with agar, 15 (BTL)). For the selection of recombinant strains bearing *HPHex* (JME4580 plasmid) or *NATr* (KO cassettes) dominant selection marker genes, hygromycin B (Sigma-Aldrich) at 250 mg L^−1^ or nourseothricin (Sigma-Aldrich) at 400 mg L^−1^ was supplemented to YPD medium (liquid or solidified). Liquid cultures were shaken at 220 rpm.

Bacterial strains were routinely maintained at 37 °C (liquid cultures were shaken at 220 rpm) in LB medium (g L^−1^: Bacto-peptone, 10 (BTL); yeast extract, 5 (BTL); NaCl, 10 (POCH); solidified with agar, 15 (BTL)) supplemented with ampicillin (Sigma-Aldrich) (100 mg L^−1^) or kanamycin (A&A Biotechnology, Gdynia, Poland) (40 µg L^−1^), as required.

All the strains were deposited as 15% glycerol stocks at − 80 °C for long-term storage.

### Basic molecular biology protocols

Standard molecular biology protocols were used in this study (Sambrook and Russell 2001). All oligonucleotides used here are listed in Supplementary Table S1C. References to backbone plasmids used in this study are given in Supplementary Table S1B. DNA fragments to be cloned were amplified using Phire Hot Start II DNA Polymerase (Thermo Fisher Scientific, Waltham, USA). Colony PCRs were conducted using RUN DNA Taq polymerase (A&A Biotechnology). Plasmid DNA isolation, agarose gel extraction, genomic DNA extraction, and post-reaction purification were all conducted using an appropriate kit from A&A Biotechnology. Restriction enzymes *Bsm*BI and *Bsa*I and T4 DNA ligase were purchased from NEB (New England Biolabs Ltd, Ipswich, USA) and used according to the manufacturer’s instructions. *Not*I and *Bgl*II endonucleases were purchased from Thermo Fisher Scientific. The preparation of competent cells and transformation of *E. coli* strains was conducted according to a standard heat shock protocol (Sambrook and Russell 2001). *Y. lipolytica* strains were transformed using the lithium acetate transformation protocol (Chen et al. 1997).

### Construction of Y. lipolytica deletant strains (KO-TFx)

#### Design and construction of deletion cassettes

The deletion cassette was designed on a GoldenGate scaffold used previously (Celińska et al. 2017; Larroude et al. 2018), limited to three fragments cloning: (i) ARM up, (ii) *NATr*, and (iii) ARM down. ARM up and down are approximately 1 kbp fragments upstream and downstream of the target loci encoding specific TF, flanked with A and B, and C and M overhangs indicated in Celińska et al. (2017). The central fragment, *NATr*, encodes a dominant selection marker gene flanked with regulatory elements and B and C overhangs. The cassettes were assembled using a previous protocol for the GoldenGate reaction (Celińska et al. 2017). White colonies were verified for correctness of the assembly by PCR of adjacent elements and restriction digestion of isolated plasmids. After release from the pSB1A backbone by *Not*I digestion, the cassettes were used for the transformation of *Y. lipolytica* JMY2810.

#### sgRNA oligonucleotide design and plasmid construction

The sgRNA oligonucleotides were designed using the CRISPR design tool integrated into the Benchling platform (https://benchling.com/). Targeting regions were selected close to the center of the coding sequences to disrupt ORFs (open reading frames) by integration of the deletion cassette. sgRNA oligonucleotides were selected based on their highest efficiency scores and lowest number of off-target sites. The 20-bp-long target sequences were flanked with *Bsm*BI recognition sites and with 4-bp overhangs enabling their correct integration in the JME4580 plasmid, as described previously (Larroude et al. 2020). Prior to cloning into the recipient plasmid, the sgRNA-encoding complementary oligonucleotides were annealed by mixing equimolar amounts of the single-strand oligos, heating at 95 °C for 5 min, and incubation at room temperature for 45 min. Two µL of the diluted oligos (1:100) were mixed with 100 ng of JME4580 plasmid, 2 µL T4 ligase buffer (New England Biolabs), 1 µL *Bsm*BI (New England Biolabs), 0.5 µL concentrated T4 ligase (New England Biolabs), and water up to 20 µL. Such a reaction mixture was subjected to the following cycling profile: (5 min at 55 °C, 5 min at 16 °C) × 40, 5 min at 80 °C. The reaction was then transformed into *E. coli* DH5alpha and transformants selected on LB ampicillin agar plates. White colonies were further used for the DNA constructions propagation, plasmid isolation, and verification of their correctness by PCR and restriction digestion with *Bgl*II.

#### Co-transformation of the deletion cassettes and sgRNA-encoding episomal plasmids

*Y. lipolytica* strains deleted in one of the selected TFs were generated by co-transformation of the deletion cassette and a compatible sgRNA-encoding episomal plasmid. Approximately 500 ng of each DNA construction was used per single transformation. The deletant strain selection was conducted according to the previously described methodology (Larroude et al. 2020). Briefly, the co-transformation reactions were inoculated into 9 mL of YPD-hygromycin-nourseothricin liquid medium and cultured at 28 °C for 48 h with shaking at 150 rpm. One mL of such cultures was then transferred into 9 mL of YPD-nourseothricin medium and incubated at 28 °C for 24 h with shaking at 150 rpm to allow plasmid curing (dropping-off JME4580). Finally, the culture was diluted and plated on YPD-nourseothricin agar. Clones appearing after 48 h of incubation at 28 °C were verified for correct integration of the deletion cassettes by PCR and sequencing.

### Phenotype analyses

#### Miniaturized cultures under selected stress factors

The cultures for the OE and KO strains’ phenotype testing were performed in 24-well plates (Biologix, Shandong, China) in 1-mL working volume. Medium for the cultures was carefully adjusted to minimize background FL, provide sufficient amounts of carbon and nitrogen to avoid starvation, and maintain the pH stably throughout the culturing time. The basic medium was composed of [g L^−1^]: yeast nitrogen base, 5.1 (Sigma-Aldrich); (NH_4_)_2_SO_4_, 15, (POCH); glucose, 20 (POCH); buffered with 0.2 M maleic acid (Sigma-Aldrich) to pH 5.0 by addition of 20% NaOH (w/w) (POCH). Pre-cultures were developed in the basic medium for 18 h at 28 °C with shaking at 380 rpm in a microplate shaker-incubator (Biosan, Riga, Latvia). The main cultures were inoculated at 5% (v v^−1^).

**Fig. 1:**
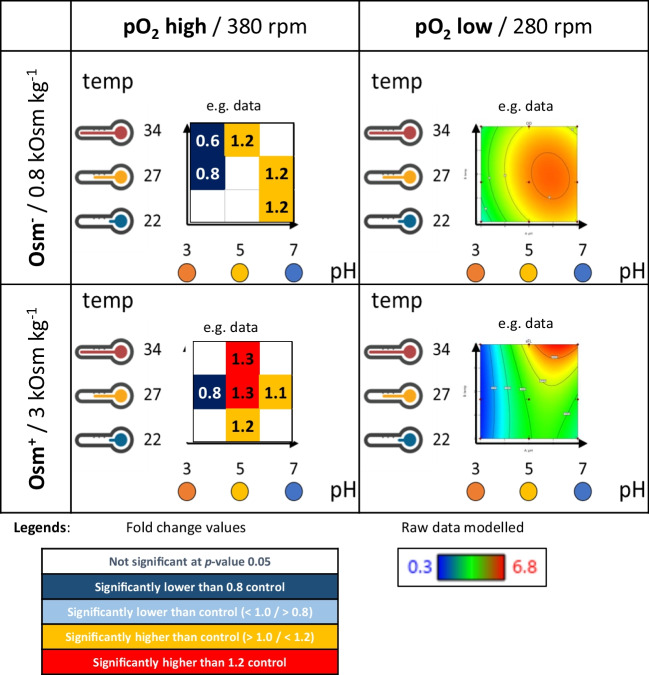
Schematic representation of experimental design and data presentation scheme of the phenotype testing. A combination of the two categoric factors (pO_2_ – oxygen availability at two levels – high 380 rpm/low 280 rpm; Osm – hyperosmolality at two levels – high 3 Osm kg^−1^/low 0.8 Osm kg.^−1^) results in the division of the design into four subpanels. Each subpanel represents data for a given response under a combination of the two categoric factors at one of the two levels. Data in the subpanels are presented as quadratic response surface (contour plot), where the temperature is plotted on the *y*-axis and pH on the *x*-axis, both ascendingly (from 22 to 34 °C and from pH 3 to pH 7). Contour lines join points of equal value. Results for the control strain are presented as contour plots solely. Data for the TF-engineered strains are presented in the form of a contour plot and a corresponding box plot (the same structure). Box plots indicate fold changes (FC) of a read parameter value for a given TF-engineered strain and the control strain under specific conditions. The two legends show color-coding of the contour plots and box plots. Values in the contour plots are color-coded from green – the lowest, through yellow, to red – the highest. Values in the box plots are color-coded from navy – significantly lower than the control by more than 20%, through blue – significantly lower than the control by < 20%, through yellow – significantly higher than the control by < 20%, to red – significantly higher than the control by 20%. The structure of the scheme of data presentation corresponds to the structure of the data presented in Figs. 2, 3, and 4

The main cultures implementing stress factors were designed to introduce two categoric stress factors: (i) oxygen availability (pO_2_) stress, by a change in shaking frequency (~ 25% reduction in rpm, from 380 to 280 rpm), (ii) hyperosmolality (Osm) stress, by the addition of sorbitol to the medium (360 g L^−1^, resulting in a 3.75-fold increase in osmolarity from 0.8 to 3 Osm kg^−1^); and two numeric stress factors: (i) three levels of cultivation temperature (22 °C, 27 °C, or 34 °C); (ii) three pH levels (pH 3.0, 5.0 or 7.0). The pH was stably maintained at the selected levels with 0.1 M maleic acid to pH 3.0 or 0.2 M maleic acid to pH 5.0 or 7.0, regulated by the addition of 20% NaOH (w w^−1^). Figure 1 illustrates the experimental setup. All the media were filter-sterilized with 0.22-μm syringe filters (Merck-Millipore, Darmstadt, Germany). The cultures were carried out for 72 h, and samples were collected at 0 h, 24 h, 48 h, and 72 h. pH and osmolarity (only high Osm variants) were additionally verified at the final time-point. All the above-mentioned variants were tested in combination, resulting in thirty-six cultivation variants for each tested strain (see Fig. 1). Cultures were performed in biological triplicates for each strain and condition.

Cultures were analyzed for cellular growth and FL from the reporter protein (RedStarII). Cell growth was measured spectrophotometrically immediately after sampling. The samples were diluted in 0.75% NaCl (POCH) to match a linear range of the methods. Absorbance was measured at 600 nm in transparent 96-well plates (Corning®Costar®, Sigma-Aldrich). FL was determined under pre-optimized ex/em settings to maximize discrimination between negative control samples (endogenous FL from cells, without RedStarII) and FL reporter-producing cells and to limit the background FL from cultivation media. FL was measured in black opaque plates (Thermo Fisher Scientific) at ex/em 554/600 nm. Both measurements were done using an Infinite M200 automatic plate reader (Tecan Group Ltd., Männedorf, Switzerland).

#### Neutral lipid storage analysis

Pre-cultures were developed in 2-mL YNB medium ([g L^−1^]: yeast nitrogen base, 1.7; (NH_4_)_2_SO_4_, 5; glucose, 20) for 23 h under 30 °C with shaking 200 rpm. The main cultures were inoculated at 5%. Two medium variants were used: (i) medium Lip^+^ of C/N ratio 150 ([g L^−1^]: glycerol, 150; (NH_4_)_2_SO_4_, 1; KH_2_PO_4_, 1, MgCl_2_x6H_2_O, 0.5), (ii) medium Lip^−^ of C/N ratio 4 ([g L^−1^]: glycerol, 20; (NH_4_)_2_SO_4_, 5; KH_2_PO_4_, 1, MgCl_2_x6H_2_O, 0.5). The main cultures were conducted for 48 h under 27 °C with shaking at 150 rpm. Nile Red (Sigma-Aldrich) reagent was diluted in dimethyl sulfoxide (DMSO) to reach 25 mg mL^−1^ and diluted to 100 µg mL^−1^. Forty-eight-hour cultures were pelleted, washed in sterile saline solution, diluted tenfold, and stained with Nile Red (working concentration 12.5 µg mL^−1^; 20 min shaking at 30 °C). Readings were done using black opaque, clear bottom 96-well plates (Thermo Fisher Scientific), using an Infinite M200 automatic plate reader (Tecan) at ex/em 488/585 nm wavelength. Control reactions without cells and without Nile Red were run simultaneously to account for non-specific background FL. Absorbance at 600 nm wavelength was measured to express the results in specific FL (sFL) units.

### Data processing and statistical analyses

All the results on cellular growth and FL are mean values ± standard deviation (from three biological replicates and technical duplicate). sFL was calculated by dividing the value for RFU (relative fluorescence units) per OD_600_ value. Fold change (FC) values were calculated by dividing the raw data for OD_600_, FL, or sFL for the OE/KO strain (*n* = 3) by corresponding results for the control strain (*n* = 3), in combination, resulting in nine FC values, out of which the mean was calculated. Statistical analyses were performed in Statistica (StatSoft-Tibco, Tulsa, USA), the analysis of variance (ANOVA) with a significance level of *p* < 0.05, preceded by Shapiro–Wilk’s and Levene’s tests to test presumptions and followed by Tukey’s HSD multiple comparisons test.

Mathematical models describing growth, FL, and sFL of the analyzed strains under the analyzed conditions were developed using response surface methodology (RSM) in Design Expert software (StatSoft). Raw results (not FC) for growth, FL, and sFL were used in the mathematical modeling. The mode of data transformation, type of model used, fitting, and significance of the model were assessed using default software settings.

FC values were used to present the experimental data as box plots. A twenty % change over the reference strain was considered a considerable change.

## Results

### Experimental plan

In this study, the experimental design covered a set of five OE strains (OE-YAP-like, OE-SKN7, OE-GZF1, OE-HSF1, OE-CRF1) and three KO strains (KO-SKN7 = *Δskn7*, KO-GZF1 = *Δgzf1*, KO-HSF1 = *Δhsf1*) that were subjected to extensive phenotype testing under thirty-six conditions. The list of engineered TFs with available functional annotations is given in Table 1. The strains were engineered to over-express (OE-)/contain an insertion (KO-) in a gene encoding a single TF (from among those identified in the *Y. lipolytica* genome (Leplat et al. 2018) and an FL reporter protein (RedStarII) to investigate the effects of the TFs manipulation on r-Prots synthesis. Selection of the five TFs was guided by initial high-throughput screens of OE strains conducted in 96-well Micro-Titer Plates (MTP) in different media formulations and under different experimental conditions (data not shown – screens in 96-well plates were prone to high variability and many intrinsic limitations). The following experimental data on the most interesting OE phenotypes guided the choice of the TFs to be deleted in the parental strain (over-expressing the RedStarII reporter).

**Table 1:** TFs engineered in this study with the available annotation

TF name	TF gene ID	UniProt	Functional annotation GRYC/UniProt if available
Yap-like TF	*YALI0D07744g*	Q6C9W7	Weakly similar to UniProt|Q9P5L6 *Neurospora crassa* related to AP-1-like transcription factor
Skn7	*YALI0D14520g*	Q6C937	Weakly similar to UniProt|P38889 *Saccharomyces cerevisiae* YHR206W (ortholog of YJR147W) Skn7 transcription factor, response regulator receiver of two components sensing
Gzf1	*YALI0D20482g*	Q6C8D8	Some similarities with UniProt|P78688 *Gibberella fujikuroi* nitrogen regulatory protein AreA
Hsf1	*YALI0E13948g*	Q6C5Z0	Some similarities with UniProt|P10961 *Saccharomyces cerevisiae* YGL073w Hsf1 heat shock transcription factor
Crf1	*YALI0B08206g*	P45815	UniProt|P45815 *Yarrowia lipolytica* YALI0B08206g Crf1 Copper resistance protein UniProt: copper resistance protein Crf1; transcriptional regulator involved in resistance to high copper concentration

The set of eight strains engineered in the TFs and a prototrophic control strain were subjected to a pre-designed experimental plan of phenotype testing under variable pH (numeric factor; “pH”), temperature (numeric factor; “temp”), medium osmolality (categoric factor; “Osm”), and oxygen availability (categoric factor; “pO_2_”; see Fig. 1). Ranges and methods of the variables implementation were first carefully adjusted in a series of preliminary studies, covering (i) minimal volume of the culture that enables reasonable repeatability between independent runs, (ii) type of buffer that covers the desired range and stably maintains the desired pH level until the end of the cultures, (iii) range of mixing rate that is sufficient/limiting growth, as desired in a specific culture variant, (iv) medium recipe that will not introduce high background FL noise in the RedStarII ex/em channel, but will support high discrimination between FL + and FL- cells, and (v) sampling time that enables capturing the differences between varying phenotypes, before any environmental factor becomes uniformly limiting to all of the strains (not resulting from the modified genotype).

**Fig. 2:**
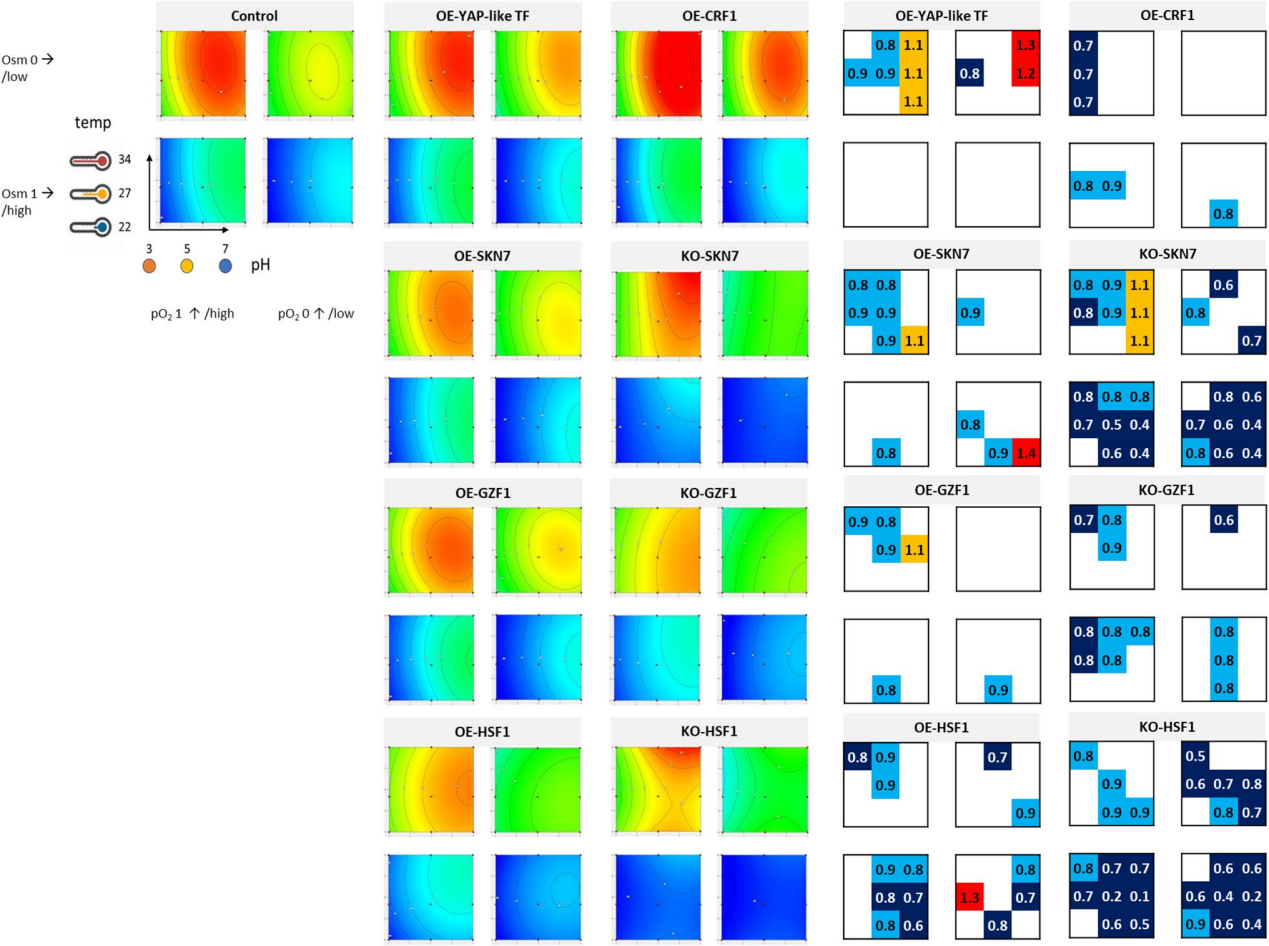
Growth of TF-engineered *Y. lipolytica* strains and a control strain under a set of the analyzed conditions. Growth is represented in the form of contour plots modeled based on raw experimental data (biological *n* = 3; technical *n* = 2) and in the form of box plots. Values in the contour plots are color-coded, as explained in Fig. 1. Numbers in the box plots correspond to fold changes (FC) of the result read for a given TF-engineered strain over the control strain under specific conditions (mean of *n* = 9). Numbers are indicated only when a result is significantly different compared to the control strain. Color code is explained in the legend of Fig. 1**.** Data presentation and reading is explained schematically in Fig. 1

**Fig. 3:**
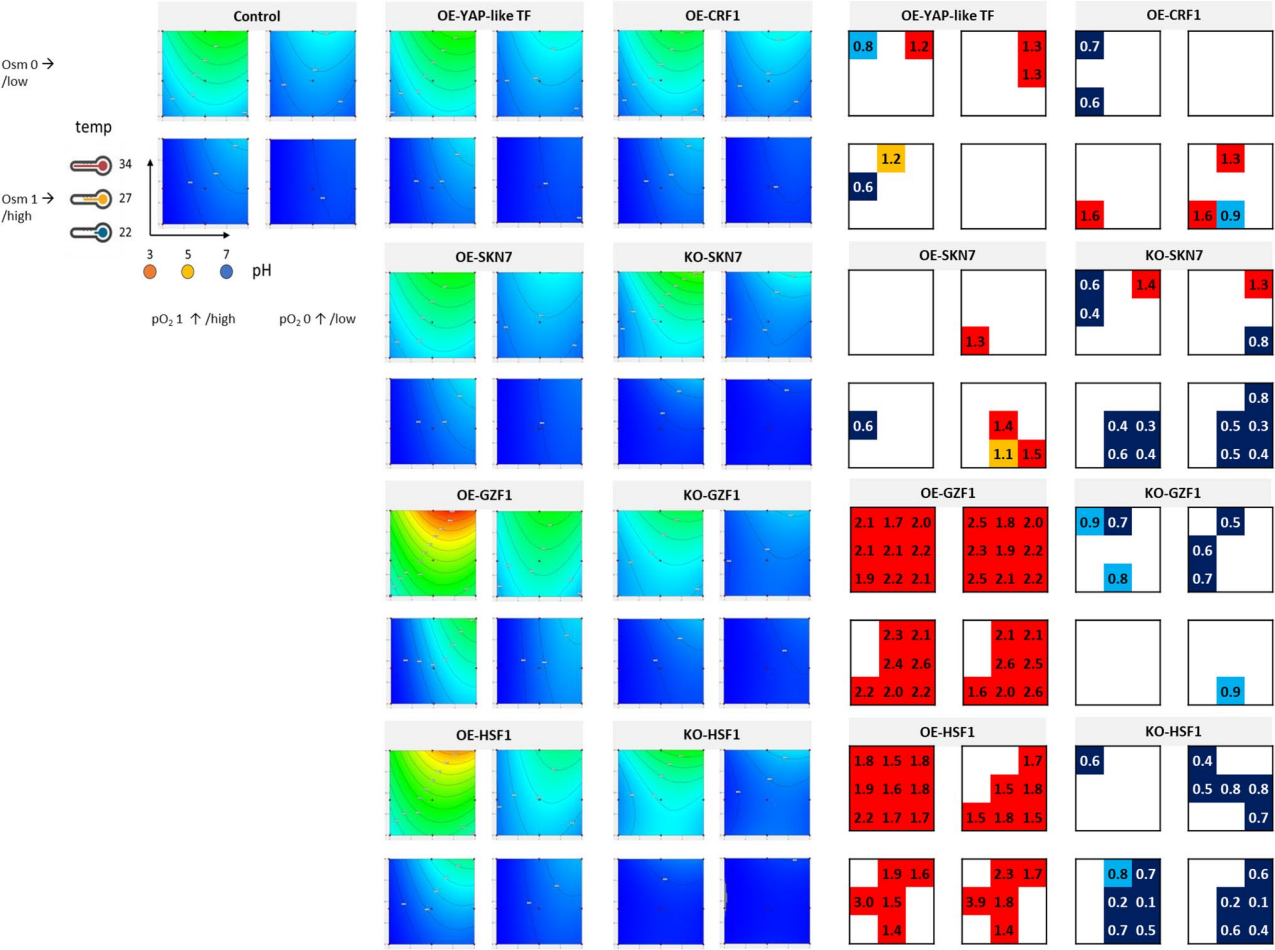
Total FL denoting total r-Prot synthesis by the TF-engineered *Y. lipolytica* strains and a control strain under a set of the analyzed conditions. FL is represented in the form of contour plots modeled based on raw experimental data (biological *n* = 3; technical *n* = 2) and in the form of box plots. Values in the contour plots are color-coded, as explained in Fig. 1. Numbers in the box plots correspond to fold changes (FC) of the result read for a given TF-engineered strain over the control strain under specific conditions (mean of *n* = 9). Numbers are indicated only when a result is significantly different compared to the control strain. Color code is explained in the legend of Fig. 1. Data presentation and reading is explained schematically in Fig. 1

**Fig. 4:**
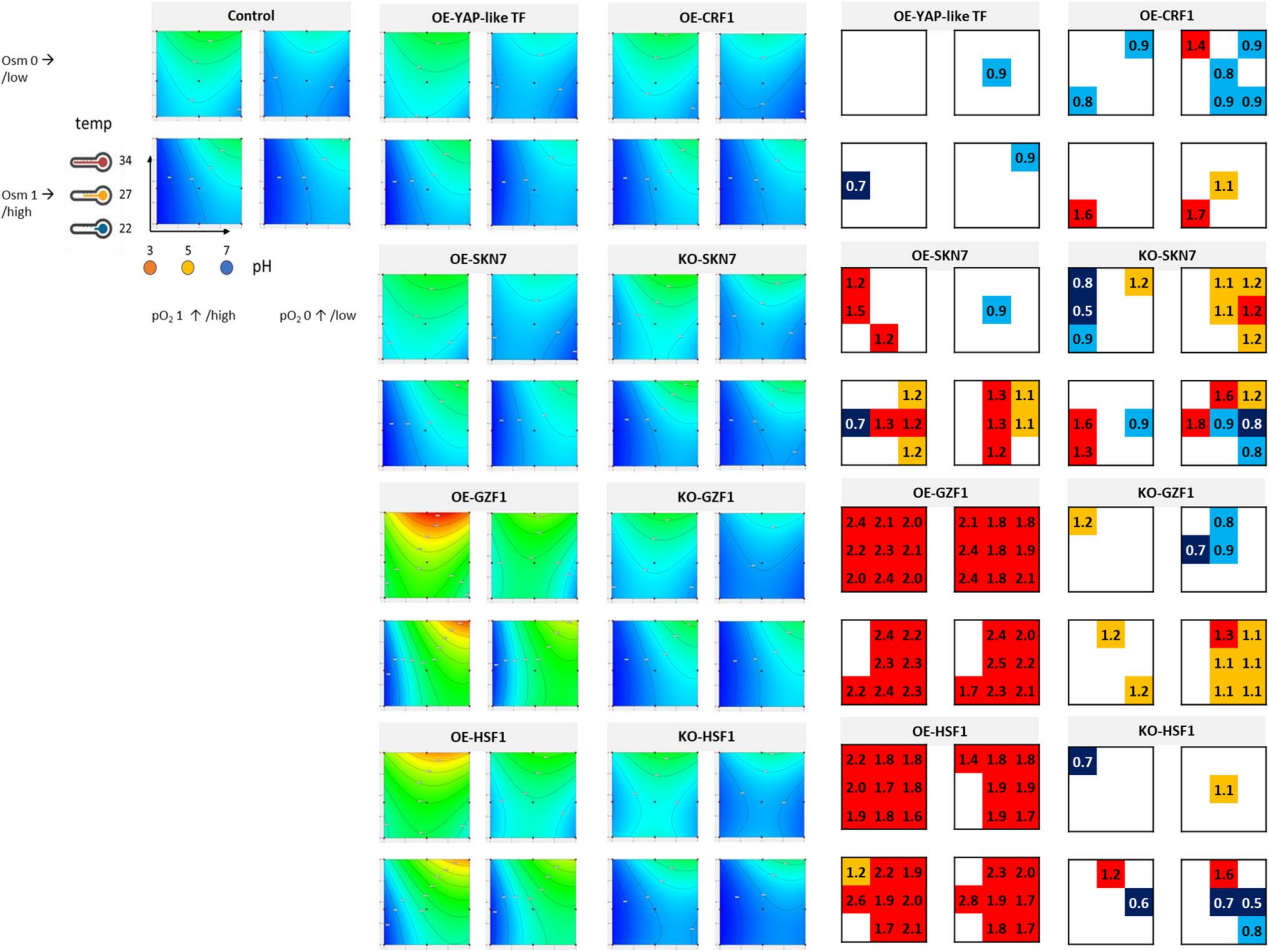
Normalized FL denoting specific r-Prot synthesis (protein synthesis capacity) by the TF-engineered *Y. lipolytica* strains and a control strain under a set of the analyzed conditions. sFL is represented in the form of contour plots modeled based on raw experimental data (biological *n* = 3; technical *n* = 2) and in the form of box plots. Values in the contour plots are color-coded, as explained in Fig. 1. Numbers in the box plots correspond to fold changes (FC) of the result read for a given TF-engineered strain over the control strain under specific conditions (mean of *n* = 9). Numbers are indicated only when a result is significantly different compared to the control strain. Color code is explained in the legend of Fig. 1. Data presentation and reading is explained schematically in Fig. 1

Growth and FL were monitored in 24-h intervals (time-point data are presented in Supplementary Fig. S1A–C). Raw data were used for mathematical modeling of the response surface illustrating observed and predicted behavior of the read parameters in a 3-dimensional space within the range of the conditions tested (Figs. 2, 3, and 4). The generated mathematical models enable evaluation of each specific parameter’s significance (temp, pH, Osm, pO_2_) for the investigated response (growth, FL, sFL), as well as the impact of the parameters interactions. These evaluations are given in Table 2. When interpreting the data from Table 2, it is important to note that the values given there correspond to the relative impact of the factor on the response (the higher value – the higher impact); but since the responses reach significantly different ranges for different strain-conditions combination (especially FL), they should not be compared between the strains. Direct comparisons of the responses from the TF-engineered strains over the control strain are shown as box plots (Figs. 2, 3, and 4). Box plots illustrate the prevalence or inferiority of the tested OE-/KO- strains over the control strain, given as FC under specified conditions (Figs. 2, 3, and 4).

**Table 2:**
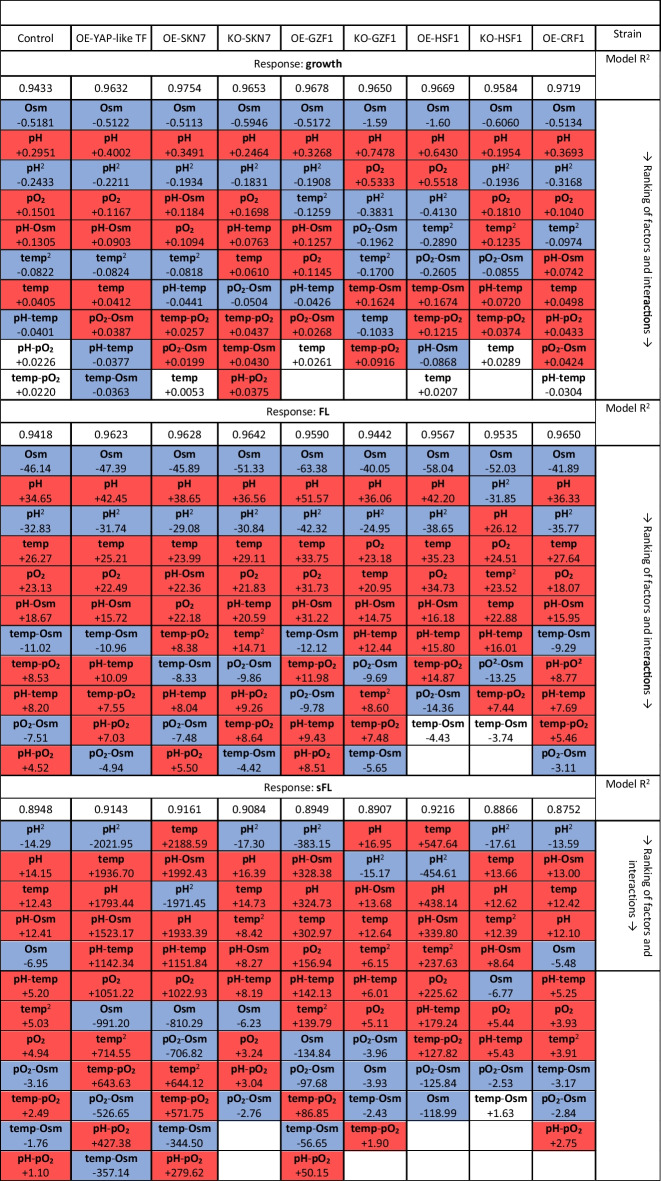
Ranking of variable factors and interactions impacting phenotype of the TF-engineered strains and the control strain

The impact of the factors and interactions was evaluated for three responses: growth, fluorescence (FL), and FL normalized per biomass (sFL; specific FL). A significant positive/negative impact of a given factor/interaction is shown in red/blue. Values indicate the relative contribution of a given factor/interaction on the response. The ranking is given from the most significant factors with the highest contribution (top) to the least significant or not significant (bottom; white fields). The positive impact is indicated by a positive value and the negative impact “ − ” as a negative value. All the values are based on developed mathematical models. All the models were significant and well-fitted to the experimental data, as indicated by the *R*^2^ values

#### Growth of the TFs-engineered Y. lipolytica strains under a set of conditions

Growth of the control strain across the conditions tested is presented in Fig. 2 as contour plots and kinetically in Supplementary Fig. S1A. As expected, the highest growth of the control strain was reached under high pO_2_ and no Osm. Under such conditions, a slight preference toward temperature > 25 °C and pH > 4.0 was observed, but the impact of pH was significantly higher than that of temp (0.2951 vs. 0.0405; Table 2). The decrease in pO_2_ at the adopted level caused significant growth limitation (impact weight 0.1501). Under Osm, the growth was significantly impeded (the highest negative impact: − 0.5181), especially when combined with low pH (significant negative impact of pH-Osm interaction, − 0.1305; Table 2).

Osm was the most detrimental parameter significantly impacting the growth of all the TF-engineered strains, followed by pH (Table 2, Fig. 2). The latter had the greatest relative impact on OE-YAP-like TF and OE-CRF1 strains’ growth (ratio pH:Osm). The modeled peaks of growth for the strains KO-HSF1 and KO-SKN7 were predicted to be at higher temperatures rather than for the remaining strains (Fig. 2), but as estimated by the model, the temp had no significant impact on the growth of KO-HSF1, and only minimal on KO-SKN7 (0.061; Table 2). In contrast, the interaction between the temp and pH gained significance for these two strains and, in addition, positively impacted their growth (+ 0.0763 and + 0.0720), in contrast to what was observed for the control strain (− 0.0401; Table 2). But generally, the temp as an individual parameter was the least significant variable impacting growth within the adopted range. Oxygen availability was particularly significant for the growth of KO-GZF1 and OE-HSF1 strains. Interestingly, the interaction between pO_2_-Osm was significant only for the TF-engineered strains (with different weights), but not for the control.

Direct comparison of growth of the TF-engineered strains and the control strain (box plots in Fig. 2) indicated that none of the TF’s engineering improved growth under Osm, except for two isolated observations for OE-SKN7 (temp 22 °C, pH 7, pO_2_ 0, Osm 1) and OE-HSF1 (temp 27 °C, pH 3, pO_2_ 0, Osm 1). Deletion of *SKN7* and *HSF1* had a detrimental impact on the strains’ resistance to Osm, irrespectively of inflicted pO_2_ stress, pH, or adopted temp. The effect of *GZF1* KO was less uniform, but also was only limiting the growth. In addition, the deletion of *HSF1* severely impacted the growth of the strain when Osm was not implemented, or even, when the conditions were favorable for growth (temp 27 °C, pH 5, pO_2_ 1, Osm 0; upper left box). In fact, most of the conducted engineering in the TFs, either OE or KO, negatively impacted the growth of the strains under the nonstressed condition. The only exception was the OE of *CRF1*, which, at the same time, exerted a specific negative impact on growth under low pH (all temp, pH 3, pO_2_ 1, Osm 0; upper left box). Interestingly, the OE of the Yap-like TF-encoding gene triggered a uniform improvement in growth under pH 7 (all temp, pH 7, pO_2_ 1/0, Osm 0; upper boxes). Correspondingly, pH had a high-weighted impact on the two OE strains’ growth (Table 2 and mentioned above). A significant, positive impact of higher pH was observed upon deletion of *SKN7*, but solely under high pO_2_ provision (all temp, pH 7, pO_2_ 1, Osm 0; upper left box).

#### Global amounts of r-Prot synthesized by the TFs-engineered Y. lipolytica strains under a set of conditions

The shape of contour plots illustrating FL from the r-Prot reporter is different from those illustrating growth (Fig. 3 vs. Figure 2), which indicates that growth was decoupled from r-Prot synthesis, even though a constitutive p*TEF* promoter was governing expression of the reporter r-Prot. All the individual variables had a significant impact on FL (Table 2). Under higher pO_2_ and no Osm infliction, a clear positive impact of temperature > 27 °C on the FL level was observed, which holds valid for all the strains (temp-pO_2_, significant interaction with positive impact for all strains). The combination of Osm and low pO_2_ severely limited the synthesis of the reporter r-Prot in all the strains (pO_2_-Osm, significant interaction with negative impact), but its relative contribution was the highest for the KO strains (position in the ranking in Table 2). Such an observation suggests that all the TFs studied here in the KO genotype contribute to maintaining r-Prot synthesis under such severe stress. Under the infliction of Osm (the most detrimental individual impact on FL; Table 2), any synthesis of the r-Prot was possible solely under pH > 5. Probably, the effort to fight against Osm and low pH was too high, and the synthesis of r-Prot was switched off (under high stress, this is one of the first biological functions arrested within the cell (Kubiak-Szymendera et al. 2021)). Interestingly, this specific interaction pH-Osm was either the most significant interaction in the ranking (Table 2) for the control strain, OE-YAP-like, OE-SKN7, OE-GZF1, KO-GZF1, OE-HSF1, and OE-CRF1, or not significant at all for KO-SKN7, KO-HSF1, even though the individual impact of Osm and pH on FL was the highest for all the strains. For the majority of the strains, the temp was the third most significant term in the developed models (Table 2), with only two exceptions, KO-GZF1 and KO-HSF1, for which pO_2_ was more significant than temp.

Direct comparison of the FL values read for the TF-engineered strains and the control strain (box plots) demonstrated significant, uniform superiority of the OE-GZF1 and OE-HSF1 over the latter in terms of the r-Prot synthesis under most of the conditions (Fig. 3). In contrast, KO-HSF1 and KO-SKN7 showed severe underperformance in terms of FL once Osm was inflicted and pH > 5. This effect was concomitant with limited growth (Fig. 2). No such pH dependency was observed for the KO-GZF1 strain (Fig. 3, Table 2). OE of *CRF1* and *SKN7* caused an increase in the FL upon several specific combinations of the stress factors and Osm (Fig. 3). For example, the OE of *SKN7* enabled maintaining of r-Prot synthesis under Osm and low pO_2_ (significant, limiting interaction); on the other hand, *CRF1* OE caused an increase in FL under low temp and pH, and Osm infliction, that sustained irrespectively of the pO_2_ factor. It is particularly interesting, considering that this combination of environmental factors is the “most stressful” from among adopted (especially pH 3 and Osm). OE of the Yap-like TF-encoding gene was concomitant with enhanced synthesis of r-Prots under pH > 5, which was associated with increased growth under these conditions (Figs. 2 and 3).

#### r-Prot synthesis capacity (sFL) of the TFs-engineered Y. lipolytica strains under a set of conditions

Contour plots illustrating calculated sFL values (Fig. 4) resemble those for FL, with several differences. Normalization per biomass highlighted the strains’ biological response in terms of r-Prots synthesis under Osm (which was not clear in the FL; Fig. 3) and substantially changed the ranking of the most significant variables (Table 2). The impact of Osm became less important, while the impact of temp gained significance, becoming the most (OE-SKN7, OE-HSF1) or second/third most (control, OE-YAP-like, KO-SKN7, KO-HSF1, OE-CRF1) significant factor. The sFL measure was relatively the least impacted by the temp for *GZF1*-engineered strains (ranking in Table 2), even though the contour plots for OE-GZF1 strain clearly indicate a positive impact of the temperature > 30 °C on sFL (Fig. 4). For these strains, pH either alone or in combination with Osm had the highest impact on sFL value. The pH-Osm followed by pH-temp remained the most significant interactions impacting sFL, as it was observed for FL (Table 2). In terms of sFL, pH-Osm became a significant interaction also for the strains KO-SKN7 and KO-HSF1, which is not true in terms of the FL for these strains (Fig. 3).

Upon direct comparison with the control strain (box plots), it appeared that OE of the *YAP-like* gene did not bring any significant positive impact on the strains’ potential toward r-Prot synthesis (Fig. 4). Any increase in the FL was a result of increased growth (Figs. 2 and 3). In contrast, OE of *GZF1* and *HSF1* brought spectacular improvement in the cells’ capacity toward r-Prots synthesis. The sFL measures for these strains were uniformly either better or comparable to the control. Surprisingly, under specific conditions (Fig. 4), KO of these genes also triggered increased sFL measure, especially seen for KO-GZF1 under Osm and KO-HSF1 under Osm (34 °C, pH 5). As in the case of FL measures, sFL readouts were comparatively higher for the OE-CRF1 strain over the control under Osm infliction, low temp, and pH (Figs. 2 and 4).

Normalization of FL per biomass enabled interesting observation of the *SKN7*-engineered strains (Fig. 4). Namely, it appeared that the relative level of sFL was significantly higher for the OE-SKN7 strain vs. the control under Osm (FC > 1.1) for 50% of the culture variants, but only when pH > 5. So, it could be concluded that OE of *SKN7* improves the protein synthesis capacity under Osm and non-stressful pH. KO of *SKN7* promoted the strains’ synthesis capacity, especially under no Osm and low pO_2_. Maintaining a high level of r-Prot synthesis capacity under Osm proves Skn7’s implication in the osmostress response.

#### Neutral lipids storage capacity of the TFs-engineered Y. lipolytica strains

**Fig. 5:**
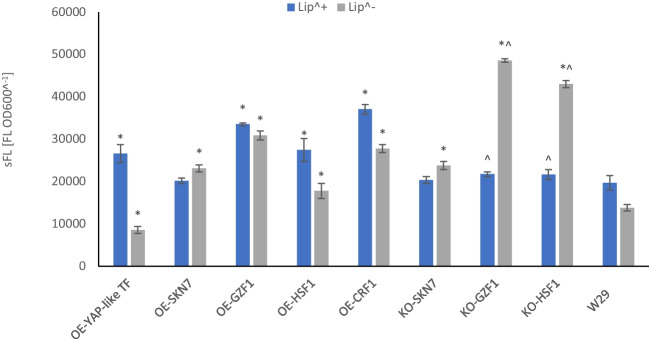
Neutral lipids storage in the TF-engineered strains and the control strain cultured in a medium promoting lipid accumulation (C/N 150; Lip^+^) and a control medium (C/N 4; Lip^−^). The cells were stained by Nile Red dye, read at 488/565 nm. Negative control accounting for non-specific FL from the cells and the dye in the polar medium was run simultaneously and considered in calculations. Results are expressed as mean values of sFL units – relative FL units (RFU) divided by absorbance at 600 nm ± SD

In an auxiliary experiment, we tested the TF-engineered strains for their capacity to store neutral lipids. To this end, the strains were cultured in two media differing in C/N ratio to promote or prevent extensive lipid accumulation (Lip^+^: C/N ratio 150, Lip^−^: C/N ratio 4). The results of lipid content expressed in sFL units are presented in Fig. 5. The most striking observation relates to strains KO-GZF1 and KO-HSF1, for which the pattern of lipid storage was inverted, as these strains accumulated significantly more lipids under Lip^−^ conditions. The same, but to a lesser extent, was observed for the *SKN7*-engineered strain. OE of any – *GZF1, HSF1*, or *CRF1* contributed to an increase in lipid content in the cell, but in these cases – according to the expected pattern (higher in Lip^+^, lower in Lip^−^).

## Discussion

This study aimed to investigate the possibility of engineering complex traits using targeted manipulation with TFs. We presumed that since TFs govern numerous molecular events due to their innate role as master regulators, their activation/disruption will presumably enable manipulation with the complex traits, eliciting the most adequate cellular response. Furthermore, since the TFs respond to different stimuli by both expression pattern and activation status (Peñalva and Arst 2002; Cornet and Gaillardin 2014), we proposed constitutive OE or KO to withdraw the TF-encoding genes from their native transcriptional regulation, combined with systematical challenging the TF-engineered strains with environmental perturbations. It was presumed that such an approach will facilitate a reliable assessment of a given TF’s involvement in the analyzed biological processes, which otherwise could be missed.

Technically, the task was indeed very challenging, as we observed that for the strictly aerobic, dimorphic yeast (with a tendency to filament and adhere to specific surfaces under stress), producing high amounts of organic acids (which is inherently associated with aerobic growth), and growing to high densities, optimizing a high-throughput culturing protocol is very demanding. Here presented methodology assures stable maintenance of the culturing conditions in terms of media acidity at the desired level and sufficient culture volume to assure phenotype expression and batch-to-batch repeatability while still maintaining high-throughput character. Equipped with such a protocol and assisted with the statistical design of experiments, we set for systematic phenotype testing of eight TF-engineered strains and a prototrophic control.

The selection of the TFs to be engineered (Table 1) was based on preliminary studies conducted according to a typical MTP-based protocol (micro-titer plate) used for *Y. lipolytica* (Leplat et al. 2015, 2018; Lazar et al. 2015) (data not shown). The least described TF studied here, the Yap-like TF (*YALI0D07744g*), shows weak similarity to an AP-1-like TF from *Neurospora crassa*. Its structure contains a basic leucine zipper (bZIP) domain that is typical for the yeast activator protein (Yap) from *S. cerevisiae*. The subfamily of Yaps is composed of eight members (Yap1-8) which are involved in environmental stress response (Rodrigues-Pousada et al. 2019). Yap1 is the major regulator of oxidative stress and is also involved in iron metabolism (like Yap5) and detoxification of arsenate (like Yap8). Yap2 is involved in cadmium stress responses, while Yap4 and Yap6 play a role in osmotic stress response. Which of the *S. cerevisiae*’s Yap1-8 is the most plausible counterpart of the *Y. lipolytica*’s Yap-like TF studied here can be only speculative. Some indications come from our auxiliary experiments, showing that OE of the Yap-like TF confers increased resistance to peroxide (like Yap1; 10 mM; assayed in drop test; not shown). In addition, since detoxification of heavy metals is impacted by external pH (via vacuolar V-ATPase activity and proton transport into the vacuole) (Eide et al. 1993; Gharieb and Gadd 1998; Ramsay and Gadd 2006), and the action of the here studied Yap-like TF (*YALI0D07744g*) is clearly pH-dependent, a weak suggestion on its similarity to either Yap1, Yap5, Yap8 or Yap2 is proposed.

Functional studies with the Yap-like (*YALI0D07744g*) TF in *Y. lipolytica* were conducted by Morales-Vargas et al. (2012). The authors observed a 3- to fivefold increase in the *YAP-like* gene transcription level during the dimorphic transition, suggesting its implication in this process. Interestingly, in that study, the dimorphic transition was induced by changing pH from 3.0 to 7.0. In the present study, we observed that the OE-YAP-like strain grew equally well or even better than the control only when pH was > 5 (1.1-fold; Fig. 2). Filamentation is also induced under pH ~ 7.0 (Gorczyca et al. 2020). Hence, based on the current data and the previous studies by Morales-Vargas et al. (2012), we propose that this TF is implicated in pH-dependent biological processes, downstream from the environmental acidity sensing/signaling. In addition, OE of the *YAP-like* gene significantly impacted lipid accumulation by enhancing it by ~ 35% under nitrogen depletion, and decreasing under Lip^−^ conditions, according to the expected pattern (Fig. 5). Previously conducted high-throughput screens including the OE-YAP-like *Y. lipolytica* strain did not show any impact of the TF-engineering on lipids accumulation (the adopted cut-off point was ± 15% over the control; (Leplat et al. 2018)). While this new observation is indeed interesting, since the capacity to synthesize r-Prot due to *YAP-like* OE was not changed (Fig. 4), this TF was not studied further for KO phenotype.

The molecular function of Skn7 (*YALI0D14520g*) in Osm stress response in *Y. lipolytica* is widely acknowledged, although no direct functional studies have been conducted yet (only studies on its upstream regulator – Hog1 (Rzechonek et al. 2018; Rzechonek et al. 2020)), while *YLSKN7* sequence is only weakly similar to an *S. cerevisiae*’s homolog (< 30%). Nevertheless, recently observed significant (fivefold) up-regulation of *YALI0D14520g* gene expression under Osm (Kubiak-Szymendera et al. 2021) supports its correct identification and its function. In the model yeast species, Skn7 is involved in osmotic and oxidative stress response. It is specifically implicated in securing chaperoning and folding capacity under stress, the onset of the oxidative stress response, and downregulation in protein synthesis. In this study, the OE of *SKN7* limited the host’s growth under multiple conditions (Fig. 2), which was the most common under no Osm and high pO_2_ (conditions favorable for growth). We are convinced that the growth limitation (in the case of OE-SKN7, but also the other OE- strains) vs. the control strain is a result of the increased metabolic burden caused by the high-level synthesis of two proteins rather than a consequence of a TF’s specific action. Our previous comparative study on high- and low-burden *Y. lipolytica* strains supports this notion (Gorczyca et al. 2022). The actual Skn7’s involvement in Osm response was highlighted in the KO-SKN7 strain exposed to Osm. Under these conditions, the growth was nearly universally abolished (irrespectively from the other conditions applied). In terms of r-Prots synthesis, Skn7’s impact was clearly marked when the FL data were normalized per biomass (sFL). In such a case, OE of *SKN7* enabled maintaining r-Prot synthesis capacity under Osm infliction (when pH was > 5; Fig. 4). The highly variable effect of *SKN7*’s KO on the sFL parameter is noted, but yet not fully clear – It promoted synthesis of r-Prots in the cell challenged with oxygen limitation, but not with Osm (Fig. 4). Manipulation with the *SKN7* gene by its KO or OE had no impact in terms of lipid accumulation capacity (Fig. 5), as both strains responded the same way under the applied conditions (Lip^+^/Lip^−^). Correspondingly, no effect of the *SKN7* OE on lipids accumulation was revealed in the high-throughput screens conducted previously (Leplat et al. 2018).

It was very interesting to see that the expected effect of *SKN7*’s KO on *Y. lipolytica*’s growth under Osm was indeed corresponding to the one elicited by KO of *HSF1* (*YALI0E13948g*). Such a significant impact of Osm on the KO-HSF1 strain was not expected (although the model confirms the significant impact of Osm on growth in KO-HSF1; Table 2), although it has a clear biological sense, considering the significance of Hsf1 to general stress response (Craig et al. 1993; Hahn et al. 2004; Verghese et al. 2012). Based on the box plots profile (Fig. 2), it can be concluded that Skn7 and Hsf1 have many overlapping functions, and they are required for growth under the majority of conditions analyzed here. OE of *HSF1* had only a negative impact on the strain’s growth, which most probably results from increased metabolic burden (as in the case of OE of *SKN7*, discussed above). Nevertheless, the key observation regarding *HSF1* was that its OE triggered a significant increase in the r-Prot synthesis (either total FL or sFL), nearly irrespectively from the applied conditions. This modification led to the highest relative increase in FL and sFL parameters under Osm infliction (up to a 3- and 3.9-fold increase over the control). Such a promoting effect was also observed in previous studies upon OE of *HSF1* in *S. cerevisiae* (Hou et al. 2012, 2013, 2014) and is now also evidenced in *Y. lipolytica*. As presented in Fig. 5, OE of *HSF1* additionally promoted the accumulation of lipids, which stays in contrast to what was observed previously in the high-throughput screens (Leplat et al. 2018), where a 28% decrease in lipids content was observed for that strain. Differences in the cultivation method and assaying conditions could account for this discrepancy.

In terms of r-Prots production, the most spectacular outcomes were reached by co-OE of *GZF1* (*YALI0D20482g*) and *HSF1* (discussed above) that uniformly improved r-Prot synthesis (either expressed in FL or sFL – Figs. 3 or 4). In the GRYC reference database (http://gryc.inra.fr/; Table 1), Gzf1 shares some similarities with *Gibberella fujikuroi*’s nitrogen regulatory protein AreA. Recent studies by Pomraning et al. (2017) shed some light on Gzf1’s function in nitrogen catabolite repression in *Y. lipolytica*. Altogether, six Gzfs are encoded in the *Y. lipolytica* genome. Those authors studied phenotypes of KO strains grown on different nitrogen sources. Structural and phylogenetic analyses showed that from among Gzf1-6, Gzf1 is the most similar to the activators of genes repressed by nitrogen catabolite repression in filamentous ascomycetes. Nonetheless, its KO did not render any aberrant phenotype, in contrast to what was observed for *Δgzf3* and *Δgzf2* strains. On the other hand, the shift in its expression level when grown on ammonium vs. peptone was the highest among *GZF*s. *GZF1* was up-regulated when the strain was cultured in the former medium and highly down-regulated when peptone was provided. Such an observation suggests that *GZF1* is highly responsive to nitrogen levels in the medium and is involved in capturing nitrogen under its limitation (activation of nitrogen assimilation genes).

As observed here, growth of the OE-GZF1 strain was not severely affected, except for occasional growth limitation, probably due to increased metabolic load (Fig. 2). In contrast, r-Prot synthesis capacity was strongly enhanced nearly irrespectively from the culturing conditions (Figs. 3 and 4). It is highly plausible that improved assimilation of nitrogen due to the *GZF1* OE could account for this result. The r-Prot synthesis capacity of the *GZF1*-engineered strains was specifically dependent on pH while less dependent on temp (Table 2). Such a specific pH dependency is related to the biological function of Gzf1 which enhances nitrogen capturing. It is known that the amino acid transporters of yeast are proton-coupled symporters, dependent on external pH. The overall proton motive force driving the internalization of amino acids is driven by the pH gradient between the outside (here pH 3 to 7) and the cytoplasm (slightly alkalic pH 7.5) (Bianchi et al. 2019). In other words, there is a direct link between ambient pH and the process of amino acid internalization. Cellular pools of amino acids impact the operation of the overexpressed *GZF1* (or redundant *GZFs* upon *GZF1*’s KO), which is the reason for the high impact of pH and pH’s interactions on OE/KO-GZF1 strains, as observed here (Table 2). Consequently, the numeric factor temp lost importance and was ranked less significant for these strains.

However, as clearly seen in many contour plots (Figs. 3 and 4), r-Prot synthesis is enhanced under higher temperatures, which is a general phenomenon, irrespective of the TFs’ engineering. This observation stays in agreement with Arrhenius’s formula for the temperature dependence of reaction rates, of course, within the tolerance range of the biological object. On the other hand, it is widely accepted that decreased temperature promotes r-Prot synthesis in multiple yeast hosts, as was also demonstrated for *Y. lipolytica* (Kubiak et al. 2021). In the following study, we explained the previous observation by dissecting r-Prots synthesis which is higher at higher temperatures (within a reasonable range) from their secretion that is promoted under decreased temperatures (Korpys-Woźniak et al. 2021). In addition, the r-Prot model used here and previously are small, fluorescent proteins with limited post-translational modifications (Korpys-Woźniak et al. 2020; Korpys-Woźniak and Celińska 2021). Typically, the temperature decrease is applied to slow down protein synthesis and enable correct folding, which is the limiting step. Since the r-Prot used here (and before) did not impose folding problems, we could observe this temperature dependency, confirmed by the high ranking of the temp factor for the sFL response by many strains in Table 2.

In contrast to what was seen previously in high-throughput screens ((Leplat et al. 2018); no impact on lipids accumulation), in this study, we observed that any genetic manipulation with *GZF1* significantly impacted the lipid accumulation capacity of the host (Fig. 5). Its OE led to enhanced lipid accumulation irrespective of the conditions (C/N) ratio. On the other hand, its disruption led to unexpected phenotype change and promoted lipids storage under the availability of nitrogen sources (conditions not favoring lipids accumulation). While without further detailed biochemical studies, it is impossible to understand how such an outcome was reached, it is concluded that manipulation with the nitrogen utilization regulator leads to changes in lipid accumulation due to failure to properly regulate/signal nitrogen metabolism or to sense and signal the intracellular nitrogen state. Similar conclusions were reached previously for *GZF2* and *GZF3* in *Y. lipolytica* (Pomraning et al. 2017). Practically, superior accumulation of lipids by the *GZF1-*engineered strain has the potential for biotechnological process development. Altogether, our current observations on *GZF1* in *Y. lipolytica* nicely complement the previously obtained data by Pomraning et al. (2017), where the functional phenotype of *Δgzf1* was not observed.

OE of *CRF1* (*YALI0B08206g*) had only a minor, negative impact on *Y. lipolytica* growth (Fig. 2), which could result from the increased metabolic burden (as discussed above for the other TFs). However, a consistent pattern of uniformly limited growth of OE-CRF1 was observed under pH 3.0 when neither low pO_2_ nor Osm stress factors were applied. Crf1 is a transcriptional regulator involved in resistance to high copper concentration, and its role in *Y. lipolytica* was confirmed by functional studies and its localization to the nucleus during growth in copper-supplemented (García et al. 2002). *CRF1* was exploited as a dominant selection marker in the genetic engineering of *Y. lipolytica* (Guo et al. 2011), confirming its function. As demonstrated by García et al. (2002), *CRF1* expression was not induced by the addition of copper to the medium, but its KO resulted in a 4- to fivefold increase in *Y. lipolytica* copper tolerance (but increased sensitivity to cadmium). It was also shown that *SOD1* (superoxide dismutase) and *MTP* (metallothionein) genes are not downstream targets of Crf1, as could be expected. The importance of the external pH in copper detoxification was discussed above for the Yap-like TF and also provides a link between the current observations on pH dependency and the known function of Crf1 in *Y. lipolytica*. In terms of *CRF1*’s OE impact on r-Prot synthesis (FL or sFL), it was interesting to see its very specific promoting effect under low pH, low temp, and Osm infliction (Figs. 3 and 4). These conditions were the harshest for the strains’ growth and r-Prot synthesis. These were also the conditions under which the beneficial effect of *HSF1* OE was lost. Therefore, this promoting effect of *CRF1* OE is particularly interesting. On the other hand, the levels of FL and sFL are generally very low under these conditions (see kinetic data in Supplementary Figs. S1B and S1C).

Our auxiliary experiments (drop tests; not shown) suggested that OE of *CRF1* slightly improved the strain’s resistance to menadione (0.25 mg mL^−1^), which is an oxidative stress inducer, but has no effect on the strain’s growth upon exposure to Osm, peroxide, or Congo Red. How these functions can be translated into the observed enhanced lipid storage capacity of the OE-CRF1 strain (Fig. 5) remains to be elucidated. One possible explanation is that OE of *CRF1* increases resistance to oxidative stress, and by these lipids (prone to oxidative damage) are accumulated at higher levels, but this statement requires further in-depth studies. Notably, a similar lipid-accumulation-promoting effect (by 85%) was observed in the previous high-throughput screens for the OE-CRF1 strain (Leplat et al. 2018).

In conclusion, our results show that Osm applied as a stress factor primarily limits growth, but its impact on r-Prots synthesis cannot be neglected (shown previously (Kubiak-Szymendera et al. 2021)). Disruption of *SKN7* and *HSF1* impairs survival under Osm, proving their function in osmostress response, but their OE is not alleviating growth under Osm infliction. Nevertheless, the OE of *SKN7* enables maintaining the r-Prot synthesis capacity under Osm. In this regard, we presume that Skn7’s action was somewhat similar to what has been recently achieved in *P. pastoris* upon *MSN4* OE (Zahrl et al. 2023). On the other hand, the elevated temperature (within the adopted range) promoted the synthesis of the easy-to-fold non-secretory r-Prots, when decoupled from growth (sFL) since growth was barely affected by the temp factor. The temperature was indeed the key parameter promoting r-Prot synthesis by OE-SKN7 and OE-HSF1, as these two TFs are intrinsically responsive to changes in thermal conditions. In the case of *GZF1*-engineered strains, pH and pH-Osm are significant for r-Prot, as they affect amino acid assimilation, and the impact of the temp factor is overbalanced. Hence, its function in nitrogen assimilation was confirmed and exploited in r-Prot synthesis engineering. OE of the Yap-like TF-encoding gene positively impacts the growth of *Y. lipolytica* under pH > 5, but not its r-Prot synthesis capacity. *Gzf1* and *Hsf1* are proposed universal r-Prot synthesis enhancers. Considering our current findings on *Y. lipolytica*’s TFs, and the results of omics studies by Lubuta et al. (2019) and Hapeta et al. (2020) on global cellular response elicited by different carbon and nitrogen sources in *Y. lipolytica*, it would be interesting to investigate the outcomes of the TFs-genetic engineering in different media formulations. Likewise, knowing the beneficial effects of increased abundance of Msn4 TF on the secretory r-Prot production in *P. pastoris* (Zahrl et al. 2023), it would be of high interest to verify if the here discovered r-Prot synthesis enhancers could also promote the secretion of r-Prots in *Y. lipolytica*.

## Supplementary Information

Below is the link to the electronic supplementary material.

Supplementary file1 (PDF 2398 KB)

## Data Availability

All data accompanying this research are presented directly in the manuscript and supplementary materials.
