# Supplementary material for: Transcription factors enhancing synthesis of recombinant proteins and resistance to stress in Yarrowia lipolytica
Source: Appl Microbiol Biotechnol. 2023 Jun 15;107(15):4853–71. doi: 10.1007/s00253-023-12607-z (PMC10344991; doi:10.1007/s00253-023-12607-z)
Supplement: Supplementary file 1 — Supplementary file1 (PDF 2398 KB) [file 253_2023_12607_MOESM1_ESM.pdf]

Applied Microbiology and Biotechnology

Transcription factors enhancing synthesis of recombinant proteins and resistance to stress in *Yarrowia lipolytica*

Supplemental Material

Maria Gorczyca[1], Jean-Marc Nicaud[2], Ewelina Celińska[1*]

[1] Department of Biotechnology and Food Microbiology, Poznan University of Life Sciences, 60-637 Poznań, Poland

[2] Université Paris-Saclay, INRAE, AgroParisTech, Micalis Institute, 78350 Jouy-en-Josas, France

*corresponding Author: ewelina.celinska@up.poznan.pl; ORCID: 0000-0001-8372-8459

Jean-Marc Nicaud, Email: jean-marc.nicaud@inrae.fr ; ORCID: 0000-0002-6679-972X

Maria Gorczyca, Email: maria.gorczyca@up.poznan.pl; ORCID: 0009-0004-7687-3753

Table S1A *Yarrowia lipolytica* strain used in this study

Strain	TF gene ID	Genotype	Phenotype	Source
JMY2810	None	Control and parental strain; derived from JMY2566 by integrating an empty overexpression cassette at the *URA3*-zeta locus	ΔAEP, ΔAXP, suc+, ura+, leu+, intracellular RedStarII, Zeta platform	(Leplat et al. 2015; Leplat et al. 2018)
		MATa, ura3::pTEF-RedStar2-LEU2-Zeta-URA3ex-pTEF-empty, leu2-270, xpr2-322		
OE-YAP-like	*YALI0D07744g*	*MATa, ura3::pTEF-RedStar2-LEU2-Zeta-URA3ex-pTEF-YAP-like-TF, leu2-270, xpr2-322*	JMY2810, OE-*YAP-like*	
OE-SKN7	*YALI0D14520g*	*MATa, ura3::pTEF-RedStar2-LEU2-Zeta-URA3ex-pTEF-SKN7, leu2-270, xpr2-322*	JMY2810, OE-*SKN7*	
OE-GZF1	*YALI0D20482g*	*MATa, ura3::pTEF-RedStar2-LEU2-Zeta-URA3ex-pTEF-GZF1, leu2-270, xpr2-322*	JMY2810, OE-*GZF1*	
OE-HSF1	*YALI1E13948g*	*MATa, ura3::pTEF-RedStar2-LEU2-Zeta-URA3ex-pTEF-HSF1, leu2-270, xpr2-322*	JMY2810, OE-*HSF1*	
OE-CRF1	*YALI0B08206g*	*MATa, ura3::pTEF-RedStar2-LEU2-Zeta-URA3ex-pTEF-CRF1, leu2-270, xpr2-322*	JMY2810, OE-*CRF1*	
KO-SKN7	*YALI0D14520g*	*MATa, ura3::pTEF-RedStar2-LEU2-Zeta-URA3ex-pTEF-empty, leu2-270, xpr2-322, Δskn, NATr*	JMY2810, KO-*SKN7*	This study
KO-GZF1	*YALI0D20482g*	*MATa, ura3::pTEF-RedStar2-LEU2-Zeta-URA3ex-pTEF-empty, leu2-270, xpr2-322, Δgzf1, NATr*	JMY2810, KO-*GZF1*	
KO-HSF1	*YALI1E13948g*	*MATa, ura3::pTEF-RedStar2-LEU2-Zeta-URA3ex-pTEF-empty, leu2-270, xpr2-322, Δhsf1, NATr*	JMY2810, KO-*HSF1*	

OE – over-expression; KO – knock-out = deletion

Table S1B *Escherichia coli* strains and plasmids used in this study

Strain	Backbone vector	Insert	Genotype/description	Source
DH5alpha	-	-	*F– endA1 glnV44 thi-1 recA1 relA1 gyrA96 deoR nupG purB20 φ80dlacZΔM15 Δ(lacZYA-argF)U169, hsdR17(rK–mK+), λ–*	Thermo Fisher Scientific (Waltham, MA, USA)
JME4580	pSB1A	HPHex_CrisprCas9-yl_RFP: CAS9 vector with hygromycinex marker; for gRNA cloning using GoldenGate	DH5alpha, episomal plasmid indicated	https://www.addgene.org/129659/
-	pCR blunt II TOPO	-	Subcloning of amplified fragments and annealed oligonucleotides in DH5alpha	Thermo Fisher Scientific (Waltham, MA, USA)
GGE029	pSB1A3-GB3	GB3 – red chromogenic protein	DH5alpha, episomal plasmid indicated	(Celińska et al. 2017)
GGE0185	JME4580	sgRNA for *GZF1*		This study
GGE0186	JME4580	sgRNA for *HSF1*		
GGE0187	JME4580	sgRNA for *SKN7*		
GGE0208	pSB1A	ARM-UP-*NATr*-ARM-DWN_*GZF1*		
GGE0209	pSB1A	ARM-UP-*NATr*-ARM-DWN_*HSF1*		
GGE0210	pSB1A	ARM-UP-*NATr*-ARM-DWN_*SKN7*		

Table S1C Oligonucleotides designed and used in this study

Name	Sequence	Description/purpose
GGP_M_*NATr*_B_F	GGTCTCTAGGT TAGGGATAACAGGGTAAT	*NATr* amplification and cloning
GGP_M_*NATr*_C''_R	GGTCTCT CCGT ATTACCCTGTTATCCCTA	
YALI0D14520g_*SKN7* 17-39 UParm_Fw	GGTCTCt GCCT GCGGCCGC TGGCCAGGATCTTGAAGCCTACA	*YALI0D14520g_SKN7* UP arm amplification from genomic DNA and cloning in pSB1A
YALI0D14520g_*SKN7* 1388-1409 UParm_Rv	GGTCTCTACCT GTTTCAGGTCGTGCAGCTCGTC	
YALI0D14520g_*SKN7* 1790-1811 DWNarm_Fw	GGTCTCT CCGT GACTCCAATGGCAAGGCCCATC	*YALI0D14520g_SKN7* DWN arm amplification from genomic DNA and cloning in pSB1A
YALI0D14520g_*SKN7* 3135-3157 DWNarm_Rv	GGTCTCTCGCA GCGGCCGC TCCGTGGATCTAAAAAGAGGGGA	
YALI0D14520g_*SKN7* 1492-1511 sgRNA_1+	CCGGTTCGAGAGACGGATTCCGGGTCGGCGCAGGTTGGGAAAGCCTTAAGAAGAAGGGTTTTAGCGTCTCTGAGCTAG	*YALI0D14520g_SKN7* sgRNA-encoding oligonucleotide to be cloned in JME4580
YALI0D14520g_*SKN7* 1492-1511 sgRNA_1-	CTAGCTCAGAGACGCTAAAACCCTTCTTCTTAAGGCTTTCCCAACCTGCGCCGACCCGGAATCCGTCTCTCGAACCGG	
YALI0D20482g_*GZF1* 12-33 UParm_Fw	GGTCTCt GCCT GCGGCCGC TGATAACGATGACACCGACTCG	*YALI0D20482g_GZF1* UP arm amplification from genomic DNA and cloning in pSB1A
YALI0D20482g_*GZF1* 971-992 UParm_Rv	GGTCTCTACCT CAGTTTGTGCACGACGTAGGCT	
YALI0D20482g_*GZF1* 1210-1231 DWNarm_Fw	GGTCTCT CCGT ACCGCGTCCACAGCCACTCGCA	*YALI0D20482g_GZF1* DWN arm amplification from genomic DNA and cloning in pSB1A
YALI0D20482g_*GZF1* 1836-1851 DWNarm_Rv	GGTCTCTCGCA GCGGCCGC CCGACATTTCCGACTGGCGATA	
YALI0D20482g_*GZF1* 1077-1096 sgRNA_1+	CCGGTTCGAGAGACGGATTCCGGGTCGGCGCAGGTTGACAGAGGCCGGACAACTCCGGTTTTAGCGTCTCTGAGCTAG	*YALI0D20482g_GZF1* sgRNA-encoding oligonucleotide to be cloned in JME4580
YALI0D20482g_*GZF1* 1077-1096 sgRNA_1-	CTAGCTCAGAGACGCTAAAACCGGAGTTGTCCGGCCTCTGTCAACCTGCGCCGACCCGGAATCCGTCTCTCGAACCGG	
YALI0E13948g_*HSF1* 14-35 UParm_Fw	GGTCTCt GCCT GCGGCCGC TTGTGCTTGTCCCAACCGACAC	*YALI0E13948g_HSF1* UP arm amplification from genomic DNA and cloning in pSB1A
YALI0E13948g_*HSF1* 1117-1138 UParm_Rv	GGTCTCTACCT AAAGGGGAAGTTGCCGCTGTTC	
YALI0E13948g_*HSF1* 1391-1412 DWNarm_Fw	GGTCTCT CCGT CTGTCGGACTTCACACCCTCCA	*YALI0E13948g_HSF1* DWN arm amplification from genomic DNA and cloning in pSB1A
YALI0E13948g_*HSF1* 2133-2155 DWNarm_Rv	GGTCTCTCGCA GCGGCCGC TCCACCAACTTTCCTCGTCAACT	
YALI0E13948g_*HSF1* 1229-1248 sgRNA_1+	CCGGTTCGAGAGACGGATTCCGGGTCGGCGCAGGTTGGGTGAGTTGGTAGAGGACAGGTTTTAGCGTCTCTGAGCTAG	*YALI0E13948g_HSF1* sgRNA-encoding oligonucleotide to be cloned in JME4580
YALI0E13948g_*HSF1* 1229-1248 sgRNA_1-	CTAGCTCAGAGACGCTAAAACCTGTCCTCTACCAACTCACCCAACCTGCGCCGACCCGGAATCCGTCTCTCGAACCGG	
sg1-*HSF1*-Fw	GGTGAGTTGGTAGAGGACAG	Verification of sgRNA-encoding oligo cloning in JME4580
sg3-*SKN7*-Fw	GGAAAGCCTTAAGAAGAAGG	
sg2-*GZF1*-Fw	ACAGAGGCCGGACAACTCCG	
sgRNA-seq700bp-JME4580-Rv	CTTCGGGTGTGAGTTGACAAG	

Figure S1A. Growth of TF-engineered strains and a control strain under tested conditions (n=36). Conditions are indicated at the top of each graph (pH – acidity, temperature in °C, OA – oxygen availability + 380 rpm / - 280rpm; Osm – osmolality + 3 Osm kg^{-1} / - 0.8 Osm kg^{-1}. Medium YNB: Yeast Nitrogen Base, 5.1; $(NH_4)_2SO_4$, 15; glucose, 20; buffered with 0.2 M maleic acid. X-axis – strain. Y axis – absorbance value at OD600. Bars' colors are explained in the legends – time-points of sample collection. Bars indicate a mean value ±SD from biological triplicate. * statistically significant vs. control, ^ statistically significant KO vs. corresponding OE strain

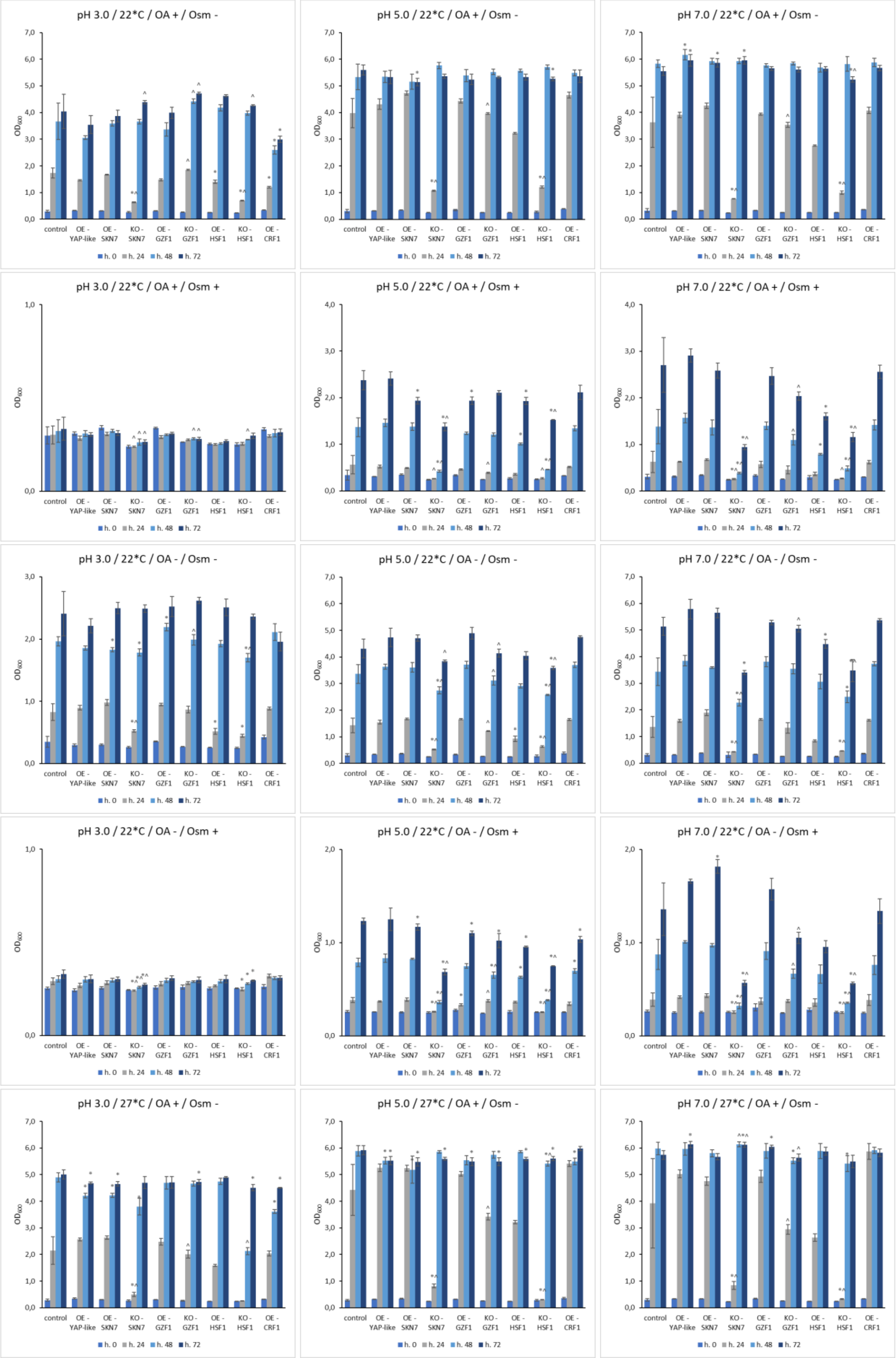

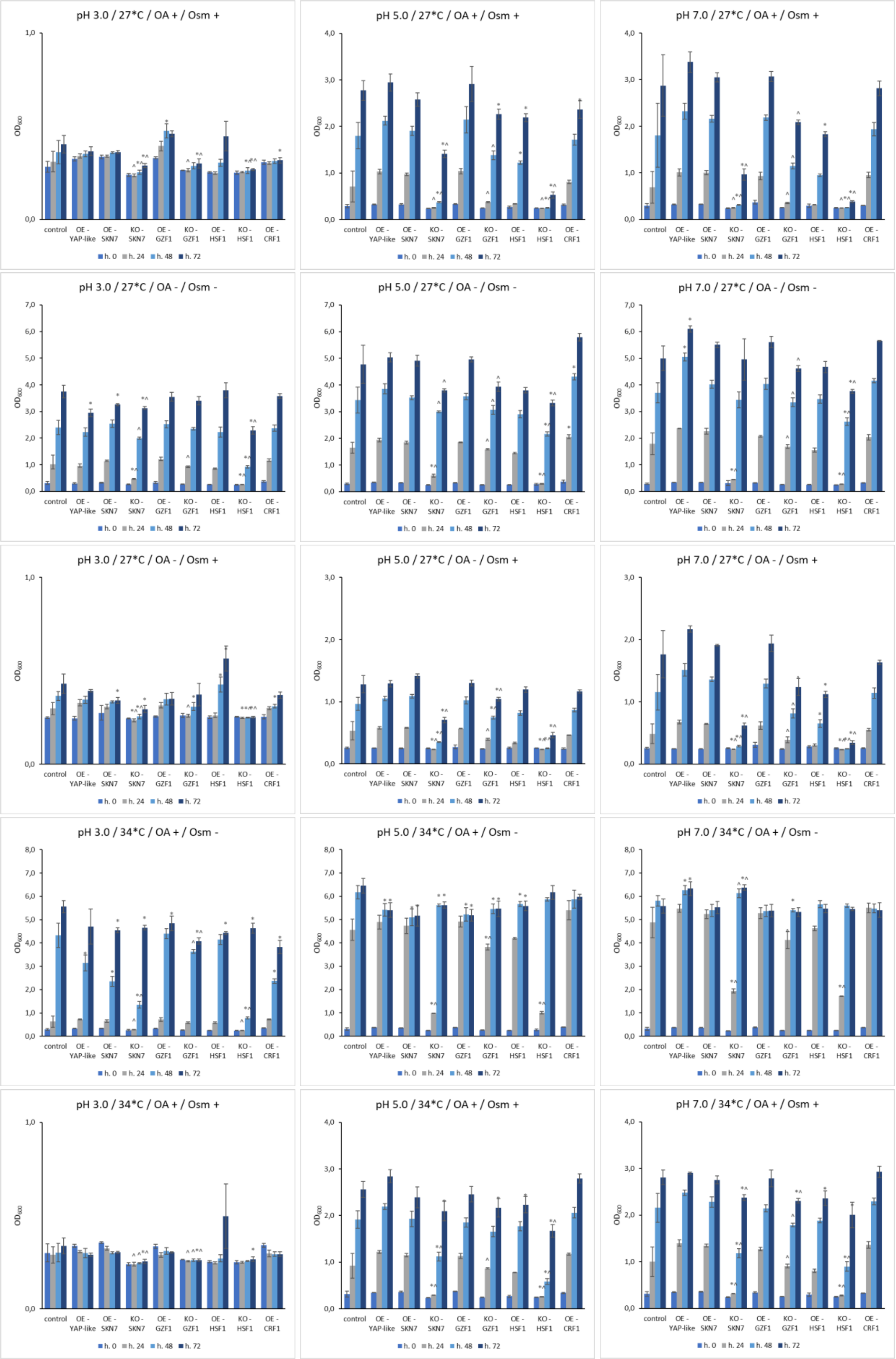
pH 3.0 / 27*C / OA + / Osm +
pH 5.0 / 27*C / OA + / Osm +
pH 7.0 / 27*C / OA + / Osm +
pH 3.0 / 27*C / OA - / Osm -
pH 5.0 / 27*C / OA - / Osm -
pH 7.0 / 27*C / OA - / Osm -
pH 3.0 / 27*C / OA - / Osm +
pH 5.0 / 27*C / OA - / Osm +
pH 7.0 / 27*C / OA - / Osm +
pH 3.0 / 34*C / OA + / Osm -
pH 5.0 / 34*C / OA + / Osm -
pH 7.0 / 34*C / OA + / Osm -
pH 3.0 / 34*C / OA + / Osm +
pH 5.0 / 34*C / OA + / Osm +
pH 7.0 / 34*C / OA + / Osm +
OD600
control
OE - YAP-like
OE - SKN7
KO - SKN7
OE - GZF1
KO - GZF1
OE - HSF1
KO - HSF1
OE - CRF1
h. 0 h. 24 h. 48 h. 72

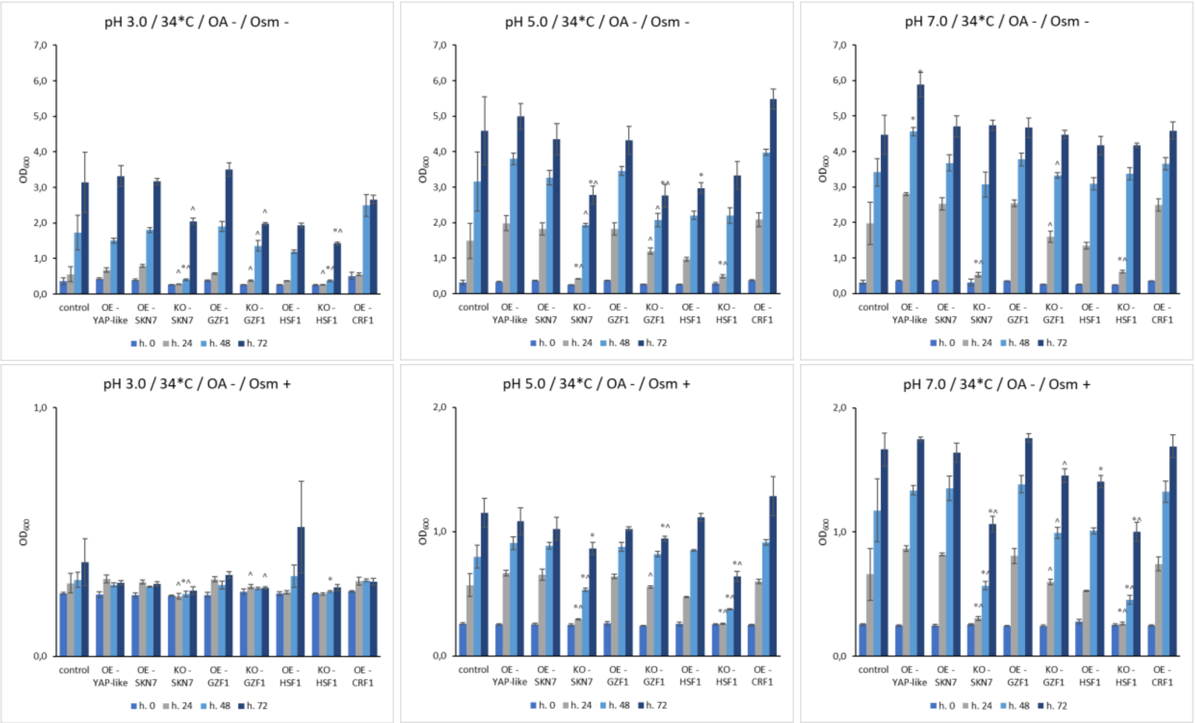
pH 3.0 / 34*C / OA - / Osm -
pH 5.0 / 34*C / OA - / Osm -
pH 7.0 / 34*C / OA - / Osm -
pH 3.0 / 34*C / OA - / Osm +
pH 5.0 / 34*C / OA - / Osm +
pH 7.0 / 34*C / OA - / Osm +
OD600
control
OE - YAP-like
OE - SKN7
KO - SKN7
OE - GZF1
KO - GZF1
OE - HSF1
KO - HSF1
OE - CRF1
h. 0
h. 24
h. 48
h. 72

Figure S1B. Fluorescence of TF-engineered strains and a control strain under tested conditions (n=36). Conditions are indicated at the top of each graph. (pH – acidity, temperature in °C, OA – oxygen availability + 380 rpm / - 280rpm; Osm – osmolality + 3 Osm kg^{-1} / - 0.8 Osm kg^{-1}. Medium YNB: Yeast Nitrogen Base, 5.1; $(NH_4)_2SO_4$, 15; glucose, 20; buffered with 0.2 M maleic acid. X-axis – strain. Y axis – Fluorescence value at ex/em 554/600nm. Bars' colors are explained in the legends – time-points of sample collection. Bars indicate a mean value ±SD from biological triplicate. * statistically significant vs. control, ^ statistically significant KO vs. corresponding OE strain

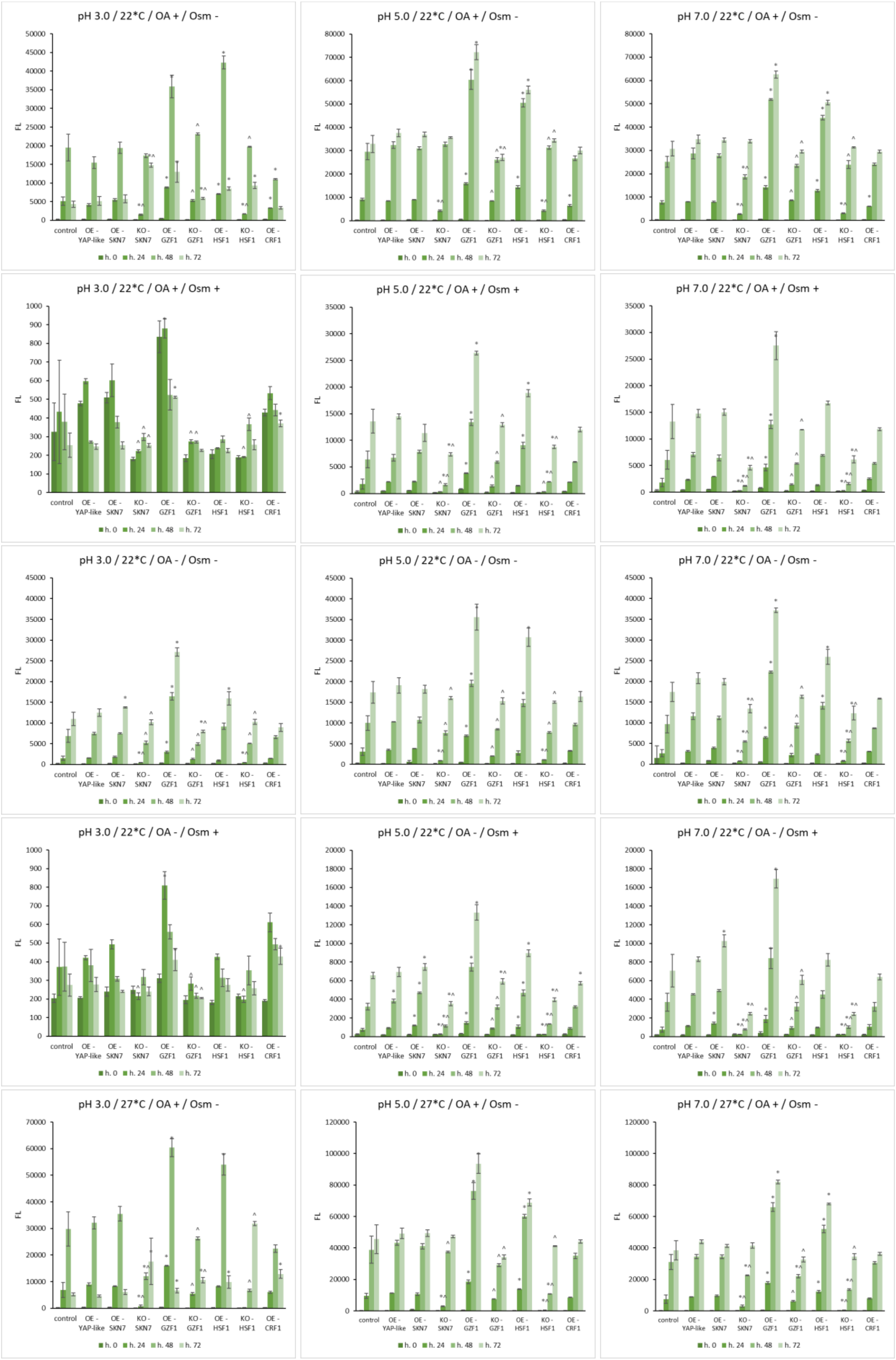

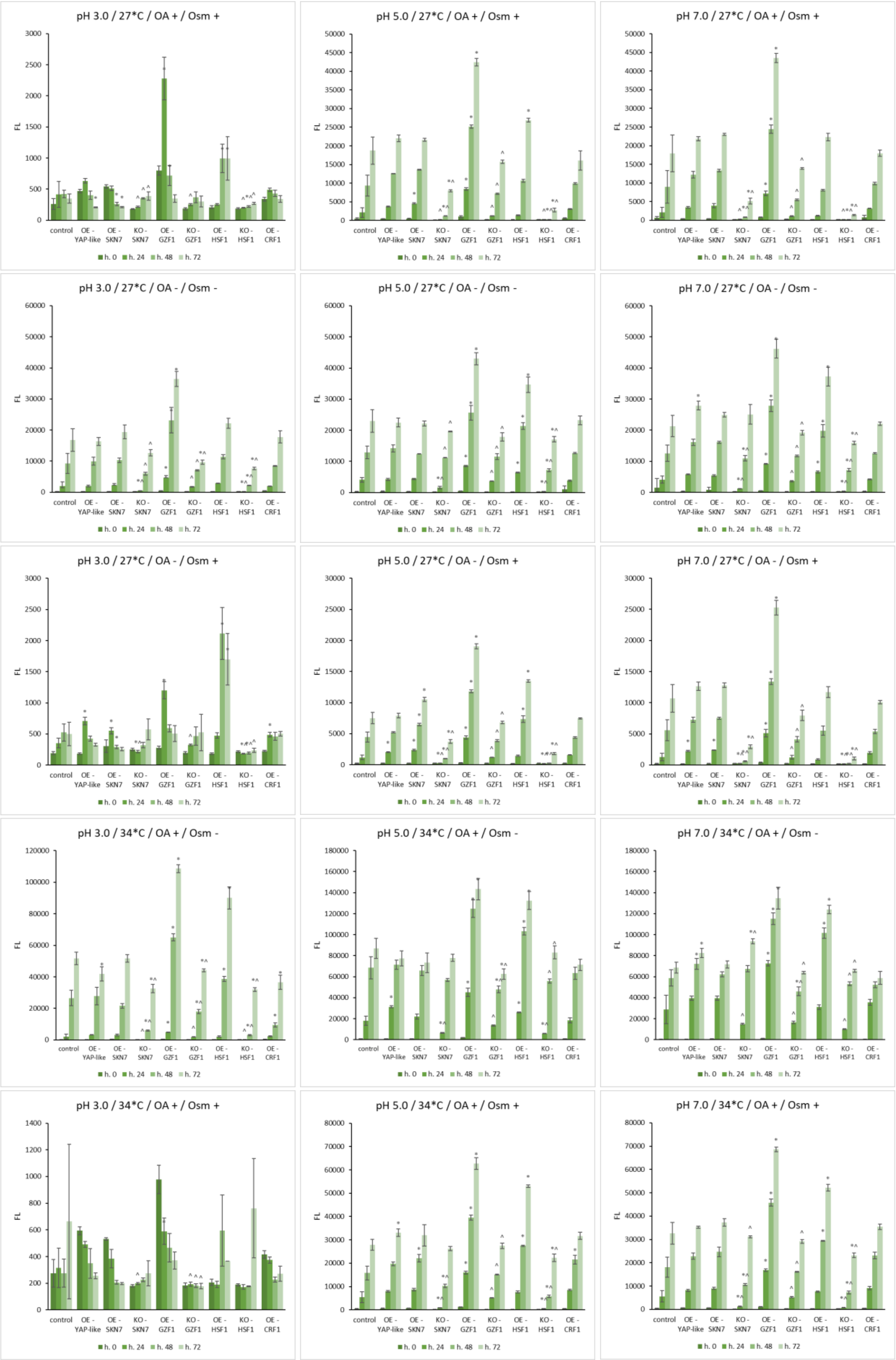
pH 3.0 / 27*C / OA + / Osm +
pH 5.0 / 27*C / OA + / Osm +
pH 7.0 / 27*C / OA + / Osm +
pH 3.0 / 27*C / OA - / Osm -
pH 5.0 / 27*C / OA - / Osm -
pH 7.0 / 27*C / OA - / Osm -
pH 3.0 / 27*C / OA - / Osm +
pH 5.0 / 27*C / OA - / Osm +
pH 7.0 / 27*C / OA - / Osm +
pH 3.0 / 34*C / OA + / Osm -
pH 5.0 / 34*C / OA + / Osm -
pH 7.0 / 34*C / OA + / Osm -
pH 3.0 / 34*C / OA + / Osm +
pH 5.0 / 34*C / OA + / Osm +
pH 7.0 / 34*C / OA + / Osm +
FL
control
OE - YAP-like
OE - SKN7
KO - SKN7
OE - GZF1
KO - GZF1
OE - HSF1
KO - HSF1
OE - CRF1
h. 0
h. 24
h. 48
h. 72

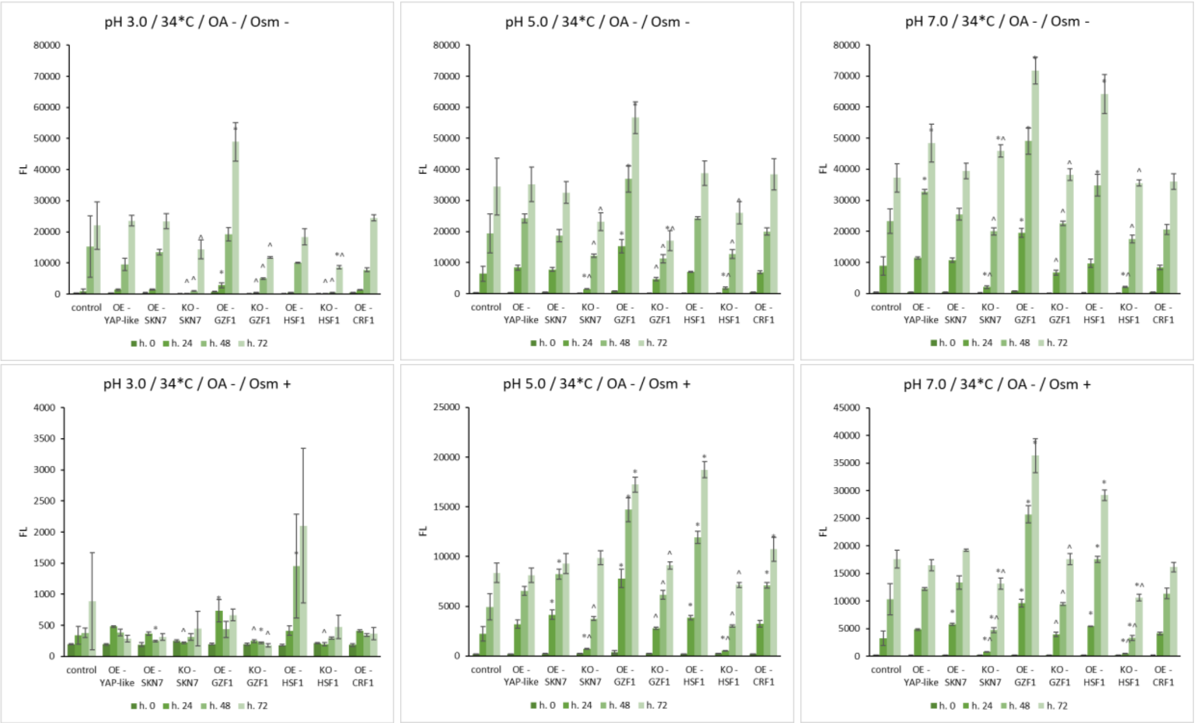
pH 3.0 / 34*C / OA - / Osm -
pH 5.0 / 34*C / OA - / Osm -
pH 7.0 / 34*C / OA - / Osm -
pH 3.0 / 34*C / OA - / Osm +
pH 5.0 / 34*C / OA - / Osm +
pH 7.0 / 34*C / OA - / Osm +
FL
control
OE - YAP-like
OE - SKN7
KO - SKN7
OE - GZF1
KO - GZF1
OE - HSF1
KO - HSF1
OE - CRF1
h. 0
h. 24
h. 48
h. 72

Figure S1C. Specific fluorescence of TF-engineered strains and a control strain under tested conditions (n=36). Conditions are indicated at the top of each graph. (pH – acidity, temperature in °C, OA – oxygen availability + 380 rpm / - 280rpm; Osm – osmolality + 3 Osm kg^{-1} / - 0.8 Osm kg^{-1}. Medium YNB: Yeast Nitrogen Base, 5.1; $(NH_4)_2SO_4$, 15; glucose, 20; buffered with 0.2 M maleic acid. X-axis – strain. Y axis – Fluorescence value at ex/em 554/600nm normalized per absorbance value at OD600. Bars' colors are explained in the legends – time-points of sample collection. Bars indicate a mean value ±SD from biological triplicate. * statistically significant vs. control, ^ statistically significant KO vs. corresponding OE strain

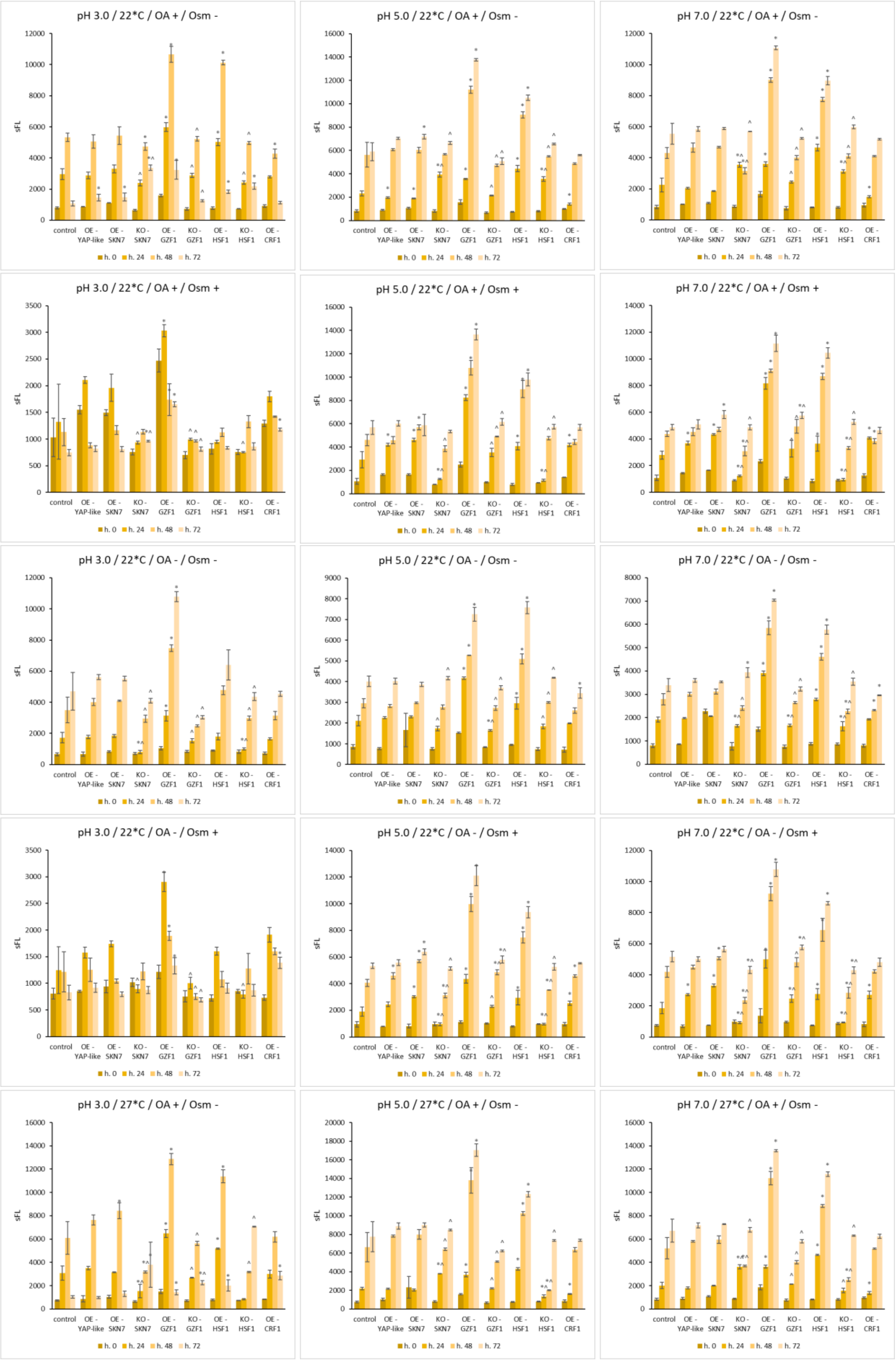

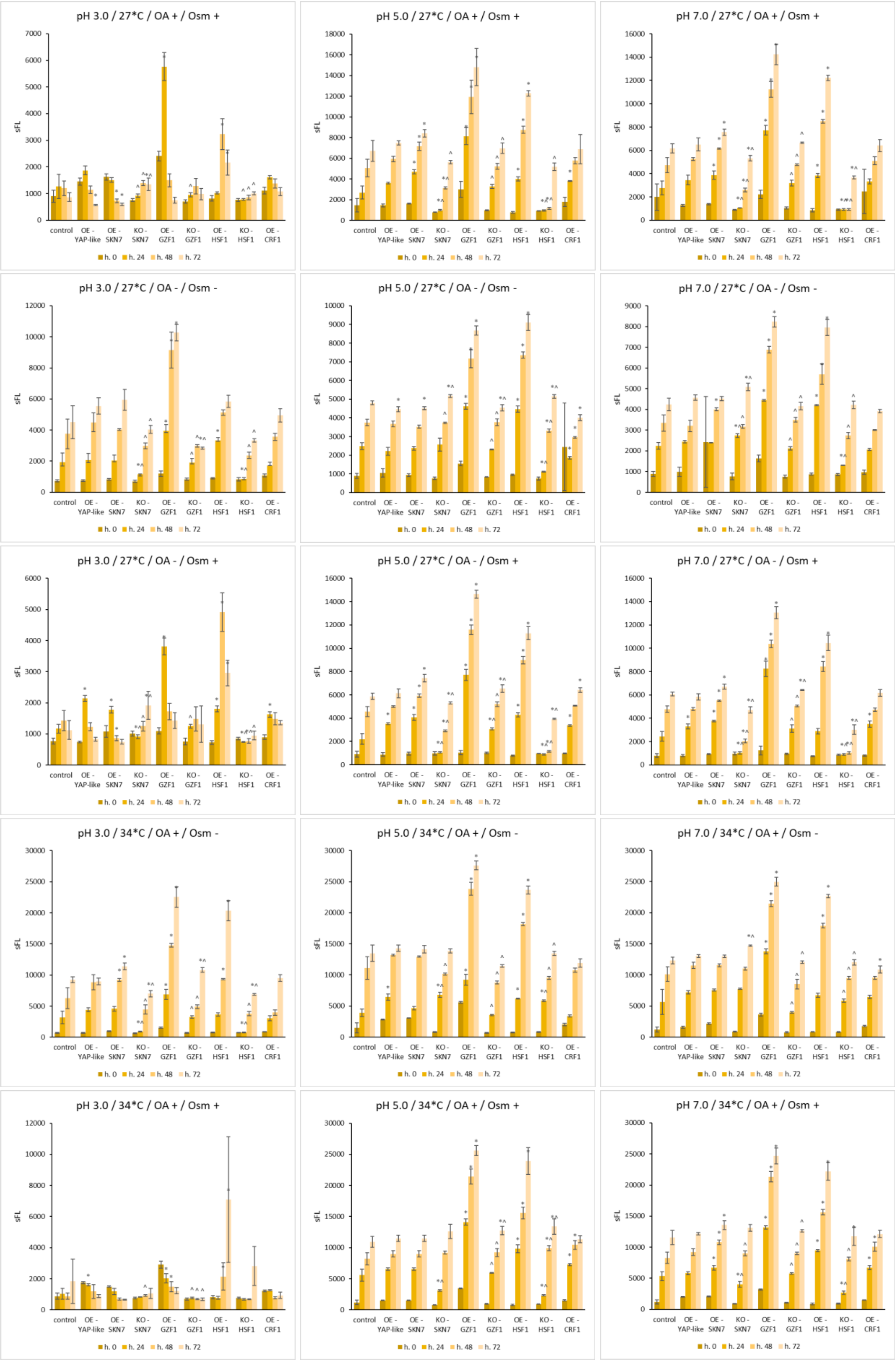

pH 3.0 / 27*C / OA + / Osm +
pH 5.0 / 27*C / OA + / Osm +
pH 7.0 / 27*C / OA + / Osm +
pH 3.0 / 27*C / OA - / Osm -
pH 5.0 / 27*C / OA - / Osm -
pH 7.0 / 27*C / OA - / Osm -
pH 3.0 / 27*C / OA - / Osm +
pH 5.0 / 27*C / OA - / Osm +
pH 7.0 / 27*C / OA - / Osm +
pH 3.0 / 34*C / OA + / Osm -
pH 5.0 / 34*C / OA + / Osm -
pH 7.0 / 34*C / OA + / Osm -
pH 3.0 / 34*C / OA + / Osm +
pH 5.0 / 34*C / OA + / Osm +
pH 7.0 / 34*C / OA + / Osm +
sFL
control
OE - YAP-like
OE - SKN7
KO - SKN7
OE - GZF1
KO - GZF1
OE - HSF1
KO - HSF1
OE - CRF1
h. 0
h. 24
h. 48
h. 72

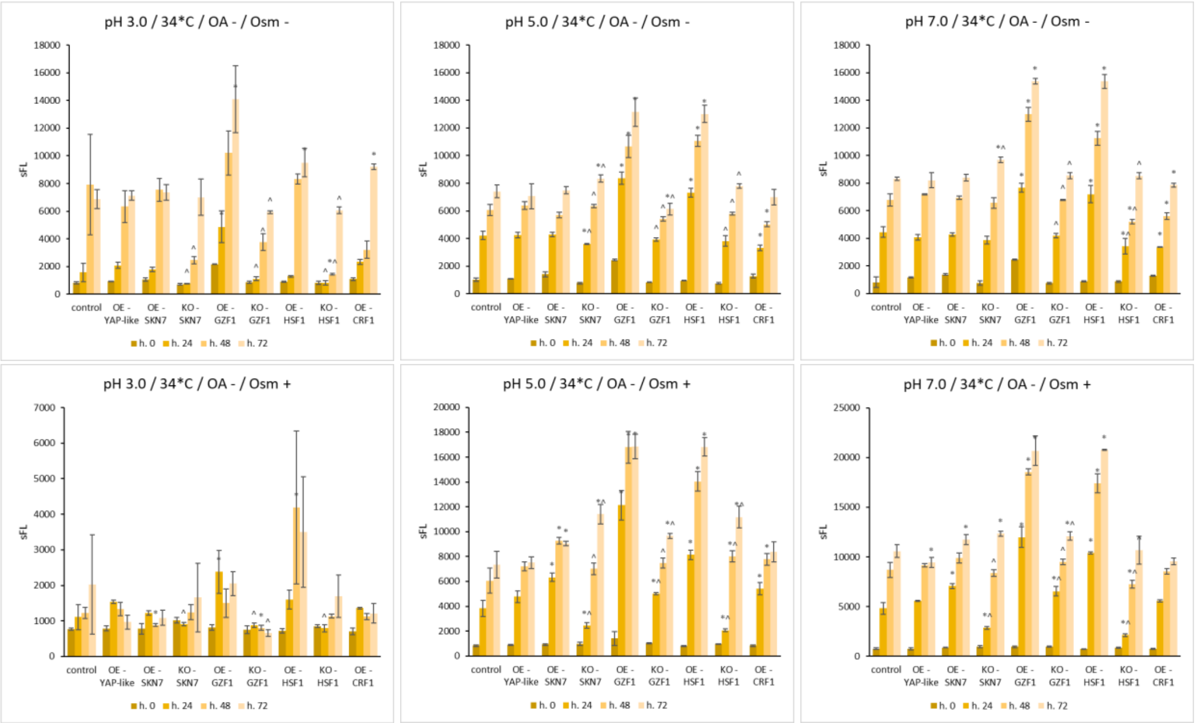
pH 3.0 / 34*C / OA - / Osm -
pH 5.0 / 34*C / OA - / Osm -
pH 7.0 / 34*C / OA - / Osm -
pH 3.0 / 34*C / OA - / Osm +
pH 5.0 / 34*C / OA - / Osm +
pH 7.0 / 34*C / OA - / Osm +
sFL
control
OE - YAP-like
OE - SKN7
KO - SKN7
OE - GZF1
KO - GZF1
OE - HSF1
KO - HSF1
OE - CRF1
h. 0
h. 24
h. 48
h. 72
